# CAF-derived exosomes drive the FGF4/SHH feedback loop by encapsulating GREM1 in non-small cell lung cancer

**DOI:** 10.1186/s10020-025-01340-0

**Published:** 2025-08-23

**Authors:** Xianqiao Wu, Wei Chen, Tianzheng Fang, Ziyuan Chen, Shuai Fang, Chengwei Zhou

**Affiliations:** 1 Department of Thoracic Surgery, The First Affiliated Hospital of Ningbo University, No.247, Renmin Road, Jiangbei District, Ningbo, 315020 Zhejiang P.R. China; 2 Health Science Center, Ningbo University, Ningbo, 315211 Zhejiang P.R. China

**Keywords:** Non-small cell lung cancer, Pre-metastatic niche, Cancer-associated fibroblast, Macrophage polarization, GREM1

## Abstract

**Background:**

One of the hallmarks of a pre-metastatic niche (PMN) is the infiltration of immunosuppressive macrophages, while the mechanism remains largely uncharted. Here, we reveal that cancer-associated fibroblasts-derived exosomes (CAF-exo) polarize macrophages towards an immunosuppressive phenotype through encapsulating Gremlin-1 (GREM1) in non-small cell lung cancer (NSCLC).

**Methods:**

CAF and normal fibroblasts (NF) were extracted from NSCLC patients, and the exosomes produced were extracted. The effects of NF- or CAF-derived exosomes on the expression of PMN-associated markers and macrophage M2 polarization markers in mouse lung and liver tissues were compared. CAF-exo-mediated macrophage phenotypic switching and immunosuppressive effects on T cells were studied in vitro. Orthotopic lung tumors were formed in mice using Lewis lung carcinoma (LLC) cells, followed by CAF-exo treatment. CAF-exo with GREM1 knockdown were used to treat macrophages or the LLC model mice. Finally, the reciprocal regulation between GREM1 and FGF4/SHH in macrophages was revealed, and rescue experiments were conducted.

**Results:**

Intravenous injection of CAF-exo induced an immunosuppressive phenotype of macrophages in lung and liver tissues, leading to PMN formation. Tumor progression promoted by CAF-exo was blocked by knocking down GREM1 in CAF-exo. CAF-exo-derived GREM1 activated FGF4/SHH signaling in macrophages, and knockdown of FGF4/SHH inhibited macrophage M2 polarization. Ectopic expression of SHH or FGF4 activated GREM1/FGF4/SHH signaling and rescued the anti-tumor effects of GREM1 knockdown in vivo.

**Conclusions:**

CAF-exo activated the positive feedback regulation of FGF4/SHH and GREM1 in macrophages by carrying GREM1, leading to the switch of macrophages to an immunosuppressive phenotype and PMN formation in NSCLC.

**Supplementary Information:**

The online version contains supplementary material available at 10.1186/s10020-025-01340-0.

## Introduction

Lung cancer is the most common oncological disease, accounting for over 2 million cases (11.6% of global cancer morbidity) and causing almost 1.8 million deaths (18.4%) per year worldwide, and can be classified as small-cell lung cancer and non-small cell lung cancer (NSCLC) (Imyanitov et al. 2021). The 5-year overall survival (OS) rate for NSCLC remains unsatisfactory, from 68% in patients with stage IB disease to 0–10% in patients with stage IVA-IVB disease because it is a metastatic disease (Duma et al. 2019). One of the first steps of metastases in lung cancer includes the formation of the pre-metastatic niche (PMN) at distant organs, which may occur even in the early phases of cancer development (Pontis et al. 2023). Extracellular vesicles (EVs), a group of cell-derived membranous structures, have been revealed to play a critical role in the formation of PMN by delivering their cargos to recipient cells (Dong et al. 2021). EVs are defined and sub-grouped based on size and cellular origin into exosomes (exo, ~ 30–120 nm, endosomal origin) and microvesicles (> 120–1000 nm, from the cell membrane) (Namee and O’Driscoll 2018). However, it remains to be elucidated how exo acts on the tumor microenvironment to promote PMN formation and what molecular mechanisms are involved in this process.

Interestingly, cancer-associated fibroblast (CAF)-derived exosomal microRNA-20a has been shown to expedite the chemoresistance of NSCLC (Shi et al. 2022). CAF, accounting for virtually 70% of the cells in tumor tissues, can shape the tumor immune microenvironment, thereby inducing tumor metastasis and immune escape (Zhang et al. 2021). However, whether CAF-exo are crucial for forming PMN in NSCLC and the underlying mechanism remains largely unexplored. Here, gremlin-1 (GREM1) was identified as a cargo of CAF-exo using combined bioinformatics analysis. GREM1 is an essential factor in the reciprocal interplay between breast cancer cells and CAF, which expedites cancer cell invasion (Ren et al. 2019). Furthermore, GREM1 overexpression increased the growth, migration, and invasion of breast cancer cells, and cells with GREM1 silencing showed reduced tumor growth rates and lung metastasis (Sung et al. 2020), which highlighted the importance of GREM1 to metastasis. In addition, a higher protein level of GREM1 in pancreatic ductal adenocarcinoma was linked to stroma formation and immunosuppression by recruiting immunosuppressive cells, including T regulatory cells, M2 macrophages, and exhausted T cells into the tumor microenvironment (Yang et al. 2022b). Tumor-associated macrophages are classified into simplistic M1 and M2 subtypes, where M1 macrophages induce an inflammatory precancerous niche, whereas their M2 counterparts are reprogrammed to release various growth factors and provide an immunosuppressive state in the tumor microenvironment (Nasrollahzadeh et al. 2020). We hypothesized that GREM1 shuttled by CAF-exo promotes the immunosuppressive state of M2 macrophages to induce the formation of PMN in NSCLC. Our study aims to explore the regulatory mechanism of CAF-exo by delivering GREM1 on macrophage-mediated PMN formation in NSCLC.

## Materials and methods

### Human tissue samples

Fresh tissue specimens from NSCLC patients (*n* = 5) and fresh whole blood from healthy volunteers (*n* = 3) were obtained between January 2022 and April 2022 at the First Affiliated Hospital of Ningbo University. Paraffin-embedded NSCLC and adjacent normal tissues from 30 patients who underwent surgical resection at the First Affiliated Hospital of Ningbo University between August 2020 and December 2021 were included for analysis.

### Immunohistochemical staining (IHC)

Paraffin-embedded human NSCLC and adjacent tissues were sectioned (5 µM). The 3% hydrogen peroxide was applied to block endogenous peroxidase activity, and heat-mediated antigen retrieval was performed using sodium citrate buffer (10 mM citrate, pH 6.0 + 0.05% Tween-20), followed by blocking with 5% goat serum. The sections were probed with primary antibodies to GREM1 (1:200, DF15419, Affinity Biosciences, Cincinnati, OH, USA), PD-L1 (1:500, 28076-1-AP, ProteinTech Group, Chicago, IL, USA), CD206 (1:1000, 18704-1-AP, ProteinTech Group), and CD8 (1:200, A11856, ABclonal, Wuhan, Hubei, China) at 4 °C overnight and with HRP-coupled goat anti-rabbit IgG (H + L) (1:200, AS014, ABclonal) for 2 h at room temperature (RT). Immunoreactive signals were detected by DAB, and hematoxylin counterstaining was performed.

Semi-quantitative determination of IHC staining was measured based on the percentage of stained cells and intensity of staining. The percentage of stained cells was scored as 0%, 0 point; 0–25%, 1 point; 25–50%, 2 points; 50–75%, 3 points; 75–100%, and 4 points. Staining intensity scores were categorized as negative, 0; weak, 1; moderate, 2; and strong, 3. The IHC score was calculated by multiplying the intensity score by the score for the percentage of positive staining (0 to 12), which was assessed by two pathologists who were unaware of the grouping, and the final score was the average of the scores of the two.

### The isolation of CAF and NF

Referring to a previous report (Shi et al. 2022), primary NF and CAF were isolated from NSCLC tissues from five patients with lobectomy. CAF were collected from the center of the tumor with a maximum diameter of < 10 mm, while NF were collected from an area of normal tissue at least 10 cm from the tumor margin. Tissue samples were cut into pieces of about 1 mm^3^ and detached with DMEM (Gibco, Carlsbad, CA, USA) containing 300 units/mL collagenase (CE12639, ChemeGen, Shanghai, China), and 100 units/mL hyaluronidase (C108116, ChemeGen), 10 ng/mL Cholera toxin (CP60310, ChemeGen) and 2% bovine serum albumin (BSA, V900933, Sigma-Aldrich Chemical Company, St Louis, MO, USA) at 37 °C for 2 h. The cell precipitate was collected by centrifugation (1000 ×*g*, 5 min) and resuspended in DMEM containing 10% FBS (Gibco). The cells were screened by a cell filter (J00100, JingAn Biological, Shanghai, China) with a pore size of 100 μm and seeded into DMEM supplemented with 1% penicillin/streptomycin, 10% FBS, and 5 µg/mL insulin and cultured at 37 °C with 5% CO_2_. The cells at passage 3 were used for subsequent experiments.

### Isolation of monocytes and T cells

Human CD14^+^ monocytes were purified from fresh whole blood donated by healthy volunteers using Dynabeads FlowComp Human CD14 Kit (11367D, Invitrogen Inc., Carlsbad, CA, USA), and human CD3 cells were purified from fresh whole blood donated by healthy volunteers by Dynabeads FlowComp Human CD3 Kit (11365D, Invitrogen). Human monocytes were maintained in RPMI-1640 (Gibco), supplemented with 10% FBS and 100 ng/mL recombinant human macrophage colony-stimulating factor for 7 days to generate macrophages. CD3^+^ T cells were activated and expanded using Dynabeads Human T Activator CD3/CD28 (11161D, Invitrogen).

In addition, monocytes were isolated from mouse peripheral blood samples using the EasySep Mouse Monocyte Isolation Kit (19861, NovoBiotechnology Co., Ltd., Beijing, China) for subsequent polarization phenotype marker assays.

### Exosome extraction and characterization

DMEM supplemented with 10% FBS was subjected to ultracentrifugation at 100,000 ×*g* for 18 h to remove exosomes. Conditioned medium (CM) was prepared by incubating NF or CAF in exosome-depleted DMEM for 48 h. CM was then centrifuged at 300 ×*g* for 10 min, 2000 ×*g* for 20 min, and 10,000 *g* for 30 min to eliminate cellular debris. After ultracentrifugation at 100,000 ×*g* for 90 min, the precipitate was resuspended in PBS and ultracentrifuged at 100,000 ×*g* for 90 min to obtain the exosome precipitate. Exosomes were quantified by examining the total protein amount using the BCA Protein Concentration Measurement Kit (Beyotime, Shanghai, China).

The exosomes were characterized under the guidance of MISEV2018 (Thery et al. 2018). Three positive protein markers (CD63, CD81, TSG101) and one negative protein marker (Calnexin) were examined by western blot analysis. The particle size was analyzed by nanoparticle tracking analysis (NTA), and transmission electron microscopy was used to observe the characteristic morphology of exosomes.

### Exosome labeling and tracking

PKH67 Green Fluorescent Cell Linker Midi Kit (MIDI67, Sigma-Aldrich) was used to label exosomes. Briefly, exosomes were incubated with 0.5 µM PKH67 dye solution at 37 °C for 5 min and rinsed twice with PBS to remove excess dye.

Four-week-old female C57BL/6J mice were procured from Vital River (Beijing, China). The animals were housed under SPF conditions with free access to food and water and underwent one week of acclimatization before the start of the experiment. Referring to a previous report (Kong et al. 2019), the distribution of exosomes was analyzed in mice. PKH67-labeled exosomes (5 µg/50 µL PBS/mouse) or control PBS were injected into C57BL/6J mice via the tail vein every other day (three times per week) for 14 days. After 14 days of exosome treatment, the mice were euthanized with sodium pentobarbital (150 mg/kg, *ip*), and lung and liver tissues were routinely paraffin-embedded and sectioned (8 μm). Tissue sections were permeabilized with 0.2% Triton X-100. Macrophages were labeled by Alexa Fluor 647 fluorescence-coupled anti-F4/80 antibody (1:50, ab307470, Abcam, Cambridge, MA, USA) overnight at 4 °C, and cell nuclei were labeled by incubation with DAPI staining solution (Beyotime) for 10 min at RT in darkness. Uptake of green fluorescent exosomes by macrophages in the tissues was observed under a fluorescence microscope. To assess retention of exosomes in tissues, mice were euthanized on days 0, 3, 6, 9, 12, and 15 after completion of the exosome injection, and liver and lung tissues were collected to assess changes in PKH67 fluorescence intensity of exosomes.

For exosome tracking in macrophages, macrophages were treated with PKH67-labeled exosomes (40 µg/mL) for 24 h and rinsed with PBS to remove unabsorbed exosomes. The cells were fixed with 4% paraformaldehyde and permeabilized with 0.3% Triton X-100. The cells were incubated with rabbit anti-CD11b antibody (1:100, A1581, ABclonal) and Alexa Fluor 647-labeled goat anti-rabbit secondary antibody (1:500, A0468, Beyotime) to label cell membranes, followed by labeling the nuclei using DAPI and observing the uptake of green exosomes under a fluorescence microscope.

### Immunofluorescence staining

Mouse lung or liver tissue Sect. (8 μm) were subjected to heat-mediated antigen retrieval using sodium citrate buffer containing 10 mM citrate (pH = 6.0) and 0.05% Tween-20, followed by blocking using 10% goat serum. The sections were primary antibodies to fibronectin (FN, 1:2000, ab268020, Abcam), lysyl oxidase (LOX, 1:900, ab174316, Abcam), MMP9 (1:5000, ab283575, Abcam), PD-L1 (1:500, 28076-1-AP, ProteinTech Group), CD206 (1:1000, 18704-1-AP, ProteinTech Group), CD8 (1:200, A11856, ABclonal), GREM1 (1:200, DF15419, Affinity Biosciences), SHH (1:500, 20697-1-AP, Proteintech Group), FGF4 (1:200, PA5-115228, Invitrogen), and F4/80 (1:500, 28463-1-AP, Proteintech Group) at 4℃ overnight. Samples were re-probed with Alexa Fluor 647-labeled goat anti-rabbit IgG at RT for 1 h in the dark and with Alexa Fluor 488-labeled anti-mouse F4/80 antibody (1:100, NB600-404AF488, Novus Biological Inc., Littleton, CO, USA) or anti-mouse CD3 antibody (1:100, 100212, Biolegend, San Diego, CA, USA). Cell nuclei were counter-stained with DAPI. The percentage of positive cells under the fluorescence microscope was calculated.

### Flow cytometry

Macrophages were resuspended in ice-cold PBS, 10% fetal calf serum, and 1% sodium azide, and the concentration of the cell suspension was adjusted to 1 × 10^6^ cells/mL. For the detection of cell surface proteins, the cells were incubated with FITC-PD-L1 (393605, Biolegend), phycoerythrin (PE)-conjugated CD206 (321105, Biolegend), and PE-CD86 (374205, Biolegend) for 1 h at 4 °C in the dark. FITC-conjugated IgG isotype (400109, Biolegend) was used as a control for PD-L1. Cells that required combined intercellular CD68 staining were fixed with 4% paraformaldehyde in ice-cold PBS for 20 min at room temperature in the dark and permeabilized with 0.1% saponin on ice for 20 min after completion of the incubation with the antibody against the surface proteins. Subsequently, the cells were incubated with FITC-CD68 (333805, Biolegend) antibody for 1 h in the dark. Detection was immediately performed by a CytoFlex S flow cytometer (Beckman Coulter, Inc., Chaska, MN, USA), and data were analyzed by CytExpert software.

### Cytokine detection

Media from each group of macrophages were centrifuged at 1000 *g* at 4℃ for 5 min, and the cell culture supernatant was collected. ELISA kits (all from FineTest, Wuhan, Hubei, China) for TNF-α (EH0302), interleukin (IL)−6 (EH0201), IL-10 (EH0173), and TGF-β1 (EH0287) were used.

### T cell and macrophage co-culture

Activated T cells were labeled by incubating with 5 µM of the fluorescent dye 5,6-carboxyfluorescein diacetate succinimidyl ester (CFSE, M5117, Abmole Bioscience Inc., Houston, TX, USA) for 15 min at RT. In the absence of exogenous cytokines, T cells and macrophages were co-cultured at a ratio of 1:5 in RPMI-1640 plus 10% FBS (Yamaguchi et al. 2022). The supernatant was collected after 3 days, and the content of interferon-γ (IFN-γ) was detected by IFN-γ ELISA Kit (CSB-E04577h, Cusabio Biotech, Newark, DE, USA), and the proliferation of CFSE-labeled T cells was analyzed by flow cytometry. T cells and monocytes-induced macrophages were derived from peripheral blood samples from the same donor.

### Cell culture and transfection

Mouse Lewis lung carcinoma (LLC) cells (CL-0140) were procured from Procell (Wuhan, Hubei, China) and were cultured in DMEM (Gibco) plus 10% FBS, 100 µg/mL streptomycin, and 100 U/mL penicillin at 37 °C with 5% CO_2_.

For transfection in cells (CAF or macrophages), GREM1/FGF4/SHH shRNA sequences (VectorBuilder, Guangzhou, Guangdong, China), GREM1 knockout plasmid (Beyotime), or overexpression plasmid of FGF4/SHH (VectorBuilder) were transfected into the cells using Lipofectamine 2000 . Subsequent experiments were performed 48 h later. A recombinant lentiviral plasmid carrying the luciferase-encoding gene (Luc) (Miaolingbio Inc., Wuhan, Hubei, China) was used to infect LLC cells, and G418 screening was utilized to obtain stably infected LCC-luc cells.

### Metastasis model of lung orthotopic implantation

Five-week-old female C57/BL6 mice were pretreated with PBS, NF-exo, CAF-exo, Exo-scramble, Exo-KD 1#, and Exo-KD 2# (exosome were not labeled with PKH67) three times per week for a total of 2 weeks, with 15 mice in each group. After 14 days, lung orthotopic tumor modeling was conducted as previously described (Hou et al. 2018). The mice were anesthetized by isoflurane inhalation and placed in right lateral recumbency. A transverse incision of 0.4–0.5 cm was made in the skin of the left chest wall near the 4th and 5th intercostal positions to expose the ribs and intercostal muscles and open the chest cavity. The left lung was fixed with forceps, and 50 µL of LCC-luc cell suspension (1 × 10^6^ cells) was administered into the lung. The incisions were sutured, and the mice were recovered under incandescent light for 10 min and returned to the animal room for observation. Mice requiring adeno-associated virus (AAV) treatment were injected with 100 µL of AAV (AAV5 carries the F4/80 promoter that specifically targets macrophages), including AAV of SHH or FGF4 overexpression vectors (AAV-SHH, AAV-FGF4) and negative control (AAV-NC, all acquired from VectorBuilder), through the tail vein using a 0.5 mL syringe with a 27G needle, followed by LCC-Luc injection. The viral titers were 10^12^ GC/mL. In preliminary assays, the use of this AAV carrying enhanced green fluorescent protein (EGFP) specifically infected macrophages (F4/80^+^) in mouse liver and lung tissues two weeks after tail vein injection into mice (*n* = 5) (Fig S1).

Bioluminescence imaging of LLC-luc tumors was performed twice weekly after tumor cell injection. Each mouse was anesthetized by intraperitoneal injection of sodium pentobarbital (35 mg/kg body weight) before observation, and Coelenterazine (M9267, AbMole) was injected intraperitoneally at 150 mg/kg. After 10 min, in vivo imaging was performed to observe the growth of the orthotopic tumors. Mouse survival was monitored, and survival rates were calculated. All surviving mice were euthanized (150 mg/kg sodium pentobarbital, *ip*) at the endpoint of the experiment (4 weeks after tumor cell injection).

### Hematoxylin-eosin (HE) staining

The HE staining kit (G1120, Solarbio, Beijing, China) was used to visualize tumor infiltration in liver tissue. Paraffin-embedded liver tissues were sectioned (5 µM), routinely dewaxed, and rehydrated. The sections were stained with a hematoxylin staining solution for 5 min and washed with distilled water. The sections were differentiated with a differentiation solution for 30 s, rinsed under tap water, and placed in an eosin staining solution for 60 s. After dehydration, clearing, and sealing of the sections, the infiltrated area of metastatic tumors in the liver was viewed under a light microscope.

### RNA extraction and qPCR analysis

Total RNA was isolated from samples to be tested using Trizol reagent (Invitrogen), and PrimeScript RT Reagent Kit (RR037B, Takara Holdings Inc., Kyoto, Japan) was used to generate cDNA from total RNA. qPCR reactions were performed on a 7500 Real-Time PCR System (Applied Biosystems, Inc., Foster City, CA, USA) using a Premix Ex Taq (RR390A, Takara). The housekeeping gene GAPDH was used as the internal control. The primers for the corresponding genes are listed in Table 1. The results were analyzed using the 2^−ΔΔCt^ method.

**Table 1:** Primer used for RT-qPCR

Common synonyms	Forward primer (5’−3’)	Reverse primer (5’−3’)
GREM1 (human)	TCATCAACCGCTTCTGTTACGGC	CAGAAGGAGCAGGACTGAAAGG
SHH (human)	CCGAGCGATTTAAGGAACTCACC	AGCGTTCAACTTGTCCTTACACC
FGF4 (human)	CGTGGTGAGCATCTTCGGCGT	GTAGGACTCGTAGGCGTTGTAG
GAPDH (human)	GTCTCCTCTGACTTCAACAGCG	ACCACCCTGTTGCTGTAGCCAA
FN (mouse)	CCCTATCTCTGATACCGTTGTCC	TGCCGCAACTACTGTGATTCGG
LOX (mouse)	CATCGGACTTCTTACCAAGCCG	GGCATCAAGCAGGTCATAGTGG
MMP9 (mouse)	GCTGACTACGATAAGGACGGCA	TAGTGGTGCAGGCAGAGTAGGA
PD-L1 (mouse)	TGCGGACTACAAGCGAATCACG	CTCAGCTTCTGGATAACCCTCG
CD206 (mouse)	GTTCACCTGGAGTGATGGTTCTC	AGGACATGCCAGGGTCACCTTT
CD8 (mouse)	ACTACCAAGCCAGTGCTGCGAA	ATCACAGGCGAAGTCCAATCCG
GREM1 (mouse)	AGGTGCTTGAGTCCAGCCAAGA	TCCTCGTGGATGGTCTGCTTCA
SHH (mouse)	GGATGAGGAAAACACGGGAGCA	TCATCCCAGCCCTCGGTCACT
FGF4 (mouse)	ACTGCAACGTGGGCATCGGATT	CACTCCGAAGATGCTCACCACG
GAPDH	CATCACTGCCACCCAGAAGACTG	ATGCCAGTGAGCTTCCCGTTCAG

*GREM1* Gremlin-1, *SHH* Sonic hedgehog protein, *FGF4* fibroblast growth factor 4, *GAPDH* glyceraldehyde-3-phosphate dehydrogenase, *FN* fibronectin, *MMP9* matrix metalloproteinase-9, *PD-L1* programmed cell death 1 ligand 1

### Western blot analyses

 Lysis buffer containing protease inhibitors (Beyotime) was used to isolate total proteins from samples to be tested on ice. The BCA Protein Concentration Measurement Kit (Beyotime) was used for protein quantification. Equal amounts of protein (20 µg) were separated by 10% SDS-PAGE and transferred to a PVDF membrane. The membranes were sealed with 5% BSA for 1 h and then incubated during the night hours at 4 °C with antibodies specific for GREM1 (1:2000, DF15419, Affinity Biosciences), SHH (1:3000, 20697-1-AP, ProteinTech Group), FGF4 (1:3000, PA5-115228, Invitrogen), GAPDH (1:5000, 10494-1-AP, ProteinTech Group), CD63 (1:5000, ab134045, Abcam), CD81 (1:5000, ab109201, Abcam), TSG101 (1:5000, ab133586, Abcam), Calnexin (1:5000, ab22595, Abcam). The membranes were incubated with HRP-coupled goat anti-rabbit IgG (H + L) (1:2000, AS014, ABclonal) at RT for 1 h. The signals of the bands were detected by ECL Western Blotting Substrate Kit (ab65623, Abcam), and the expression of the target proteins was normalized to the gray value of the internal reference band GAPDH on ImageJ software.

### Statistical analyses

Data were performed thrice and were displayed as the mean ± SEM and analyzed employing GraphPad Prism 10.4.2 (GraphPad, San Diego, CA, USA) and were compared via t-tests or analysis of variance (ANOVAs) followed by Tukey’s post hoc tests. Pearson’s correlation coefficient was used to analyze for analyzing the correlation between the expression of variables. The log-rank test was used to assess the survival rate of mice. *p* < 0.05 was the threshold of significance.

## Results

### CAF-exo from NSCLC patients promote immunosuppressive PMN formation in mice

Primary CAF and matched NF were isolated from the NSCLC tissues of five patients, and exosomes were extracted. CAF or NF-derived exo from five patients were fused isometrically, i.e., divided into two separate cohorts, CAF-exo and NF-exo, according to the type of sample from which the exo was derived. NTA and TEM were used to verify the identity of the two sets of exo, and both had a particle size of around 80–120 nm with a typical elliptical structure (Fig S2A, 2B). Western blot experiments showed that the extracted CAF-exo and NF-exo expressed CD63, CD81, and TSG101 but not the negative marker Calnexin (Fig S2C).

**Fig. 1:**
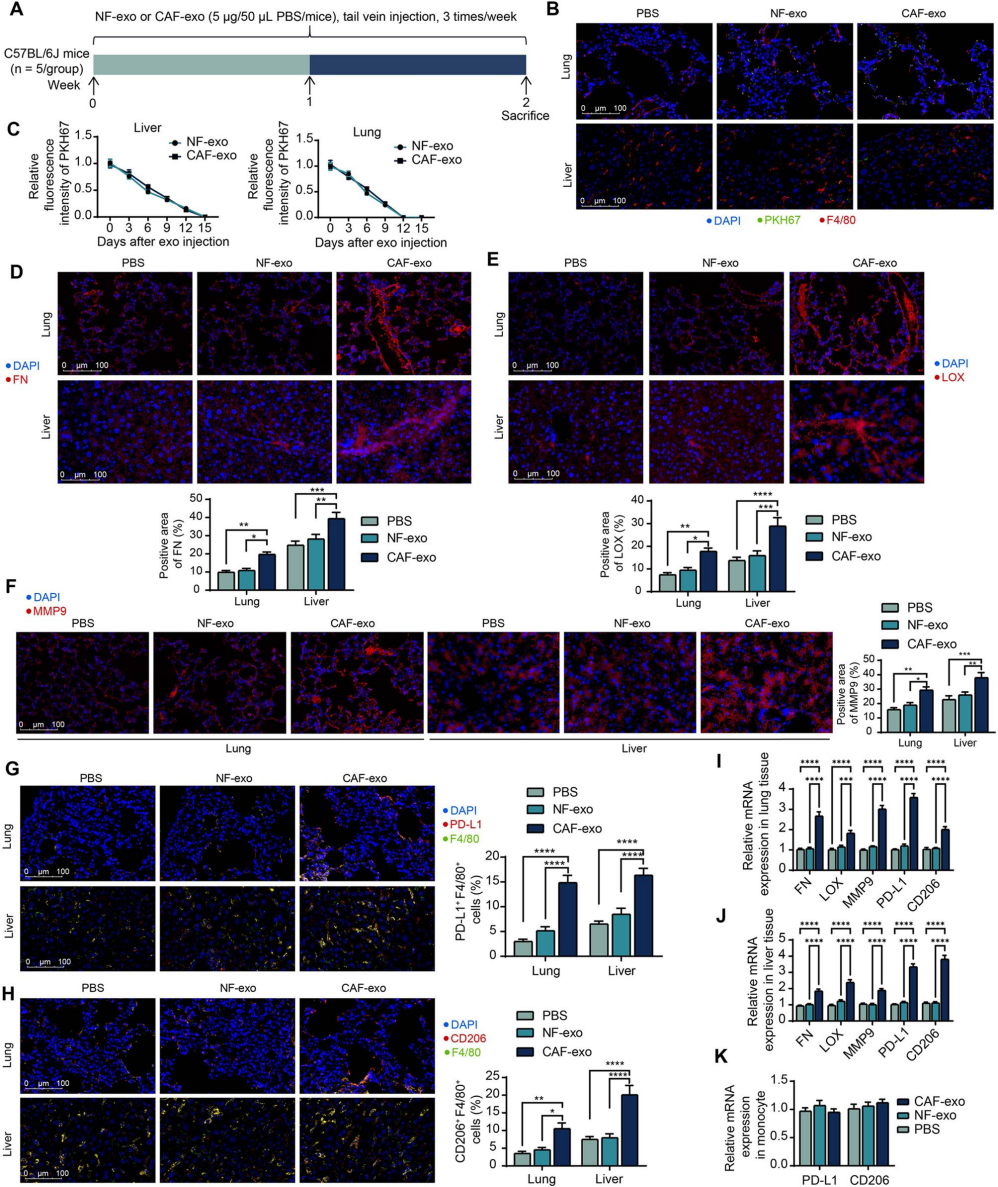
CAF-exo promotes PMN formation in mice. (**A**) Schematic for experimental design. (**B**) Phagocytosis of green fluorescently labeled exosomes by F4/80-positive macrophages was observed in lung and liver tissues. (**C**) Liver and lung tissues of mice were harvested at different time points (0, 3, 6, 9, 12, 15 days) after the completion of PKH67-labeled exosome injection, and changes in exosome fluorescence intensity in the tissues were assessed. Expression of PMN formation markers FN (**D**), LOX (**E**), and MMP9 (**F**) in lung and liver tissues of mice after CAF-exo and NF-exo injections detected by immunofluorescence. Expression of immunosuppressive macrophage markers PD-L1 (**G**) and CD206 (**H**) in F4/80^+^ macrophages of lung and liver tissues of mice after CAF-exo and NF-exo injections by immunofluorescence. Expression of FN, LOX, MMP9, PD-L1, and CD206 in lung (**I**) and liver tissues (**J**) of mice after CAF-exo and NF-exo injections by RT-qPCR. (**K**) PD-L1 and CD206 expression in mouse monocytes was examined using RT-qPCR. *N* = 5. The data are shown as mean ± SEM. *p < 0.05, **p < 0.01, ***p < 0.001, ****p < 0.001 (ANOVA)

To reveal the in vivo distribution of exo, we injected PKH67-labeled CAF-exo and NF-exo into mice via the tail vein, respectively, and mice injected with PBS served as normal controls (Fig. 1A). None of the mice showed significant discomfort or died. After 14 days, green fluorescent labeling was observed in the lungs and livers of mice receiving exosome injections, which were present in F4/80-labeled macrophages (Fig. 1B). The retention time of NF-exo and CAF-exo in liver and lung tissues was analyzed. After mice underwent an exosome injection as shown in Fig. 1A, mice were euthanized. Liver and lung tissues were collected at different time points (0, 3, 6, 9, 12, 15 d) and PKH67 fluorescently labeled exosome signals were detected in the tissues. It was observed that the exo signal was still present in the liver on day 12 after the completion of exo injection, whereas the exo signal in the lung tissues was maintained only until about day 9 (Fig. 1C). There was no significant difference in the retention of the two exo in vivo.

After exosome intervention using exosomes not labeled with PKH67, as shown in Fig. 1A, the presence of PMN markers, including FN (Fig. 1D), LOX (Fig. 1E), and MMP9 (Fig. 1F), was observed by immunofluorescence in lung and liver tissues. Significantly enhanced expression of PMN markers was found in lung and liver tissues of mice treated with CAF-exo, while NF-exo did not have this effect. We also observed an increase in the expression of the immunosuppressive markers PD-L1 and CD206 in macrophages (F4/80^+^ cells) in mouse lung and liver tissues, whereas NF-exo did not induce macrophage immunosuppressive responses at the relevant organs (Fig. 1G, H). RT-qPCR also confirmed the enhanced expression of FN, LOX, MMP9, PD-L1, and CD206 caused by CAF-exo in lung tissues (Fig. 1I) and liver tissues (Fig. 1J). In addition, monocytes were isolated from the peripheral blood of mice. The expression of PD-L1 and CD206 in monocytes was not altered by the injection of CAF-exo, nor NF-exo (Fig. 1K). This suggests that CAF-exo induced polarization in macrophages infiltrating into the tissue and not in monocytes in the peripheral blood.

### CAF-exo induce an immunosuppressive phenotype in macrophages

**Fig. 2:**
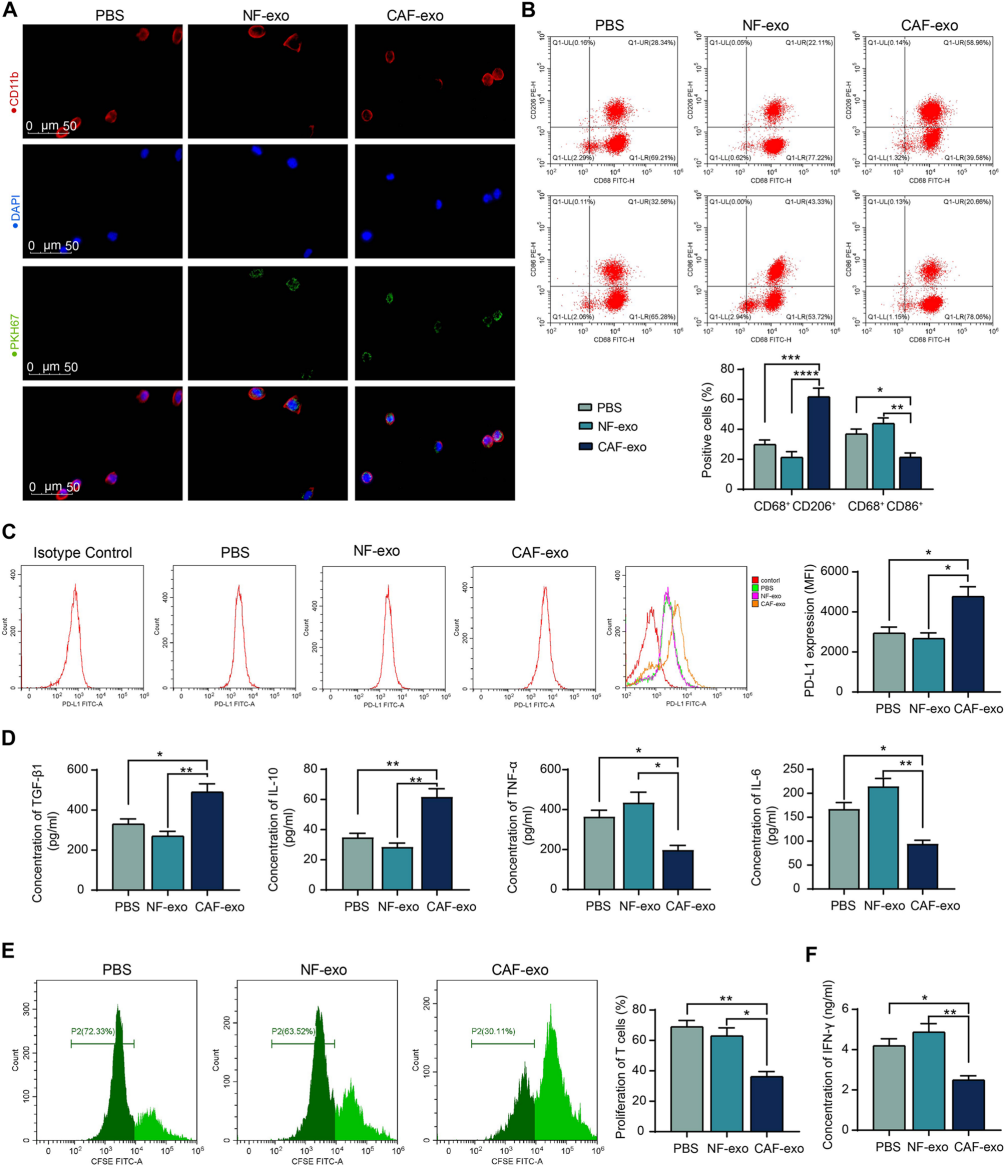
CAF-exo polarize macrophages towards an immunosuppressive phenotype. (**A**) Uptake of PKH67-labeled CAF-exo and NF-exo by macrophages. (**B**) Flow cytometry analysis of the effect of PBS, NF-exo, and CAF-exo treatment on the proportion of macrophages with M1 (CD68^+^CD86+) or M2 (CD68^+^CD206^+^) phenotype. (**C**) Detection of PD-L1 expression in macrophages by flow cytometry. (**D**) Cytokine contents released by macrophages were analyzed using ELISA kits. (**E**) T cell proliferation after co-culture with different groups of macrophages by flow cytometry. (**F**) The concentration of IFN-γ in co-culture supernatants of T cells and macrophages was analyzed using an ELISA kit. The results are representative of data from triplicate experiments. The data are shown as mean ± SEM. *p < 0.05, **p < 0.01, ***p < 0.001, ****p< 0.001 (ANOVA)

As observed in the above in vivo experiments, where CAF-exo induced macrophage immunophenotypic switching, it is not yet known whether CAF-exo directly affects macrophages derived from monocytes of human peripheral blood samples. We treated human macrophages with PKH67-labeled CAF-exo and NF-exo and observed successful macrophage uptake of exo from both sources (Fig. 2A). After that, macrophages were treated identically using CAF-exo and NF-exo that were not PKH67-labeled. Macrophage polarization was analyzed by flow cytometry detection of the intracellular marker CD68 and the surface markers CD206 and CD86. CAF-exo induced an increase in macrophage M2 polarization (CD68^+^CD206^+^) and a decrease in M1 polarization (CD68^+^CD86^+^) as compared to PBS or NF-exo treatment. NF-exo did not change the macrophage polarization relative to the PBS treatment (Fig. 2B). Meanwhile, enhanced PD-L1 expression on the surface of CAF-exo-treated macrophages was examined by flow cytometry (Fig. 2C). Immunosuppressive cytokines TGF-β1 and IL-10 were increased in the supernatants of CAF-exo-treated macrophages, while the release of pro-inflammatory cytokines TNF-α and IL-6 was attenuated (Fig. 2D).

The treated macrophages were co-cultured with T cells (they were derived from peripheral blood samples from the same donor). CFSE staining showed that co-culturing with CAF-exo-treated macrophages significantly repressed the proliferation of T cells (Fig. 2E), which occurred concomitantly with reduced IFN-γ release by T cells (Fig. 2F). By contrast, the NF-exo-treated cells did not show significant inhibition of T-cell activity (Fig. 2E, F).

### CAF-exo promotes tumor colonization and PMN formation in LLC mice

**Fig. 3:**
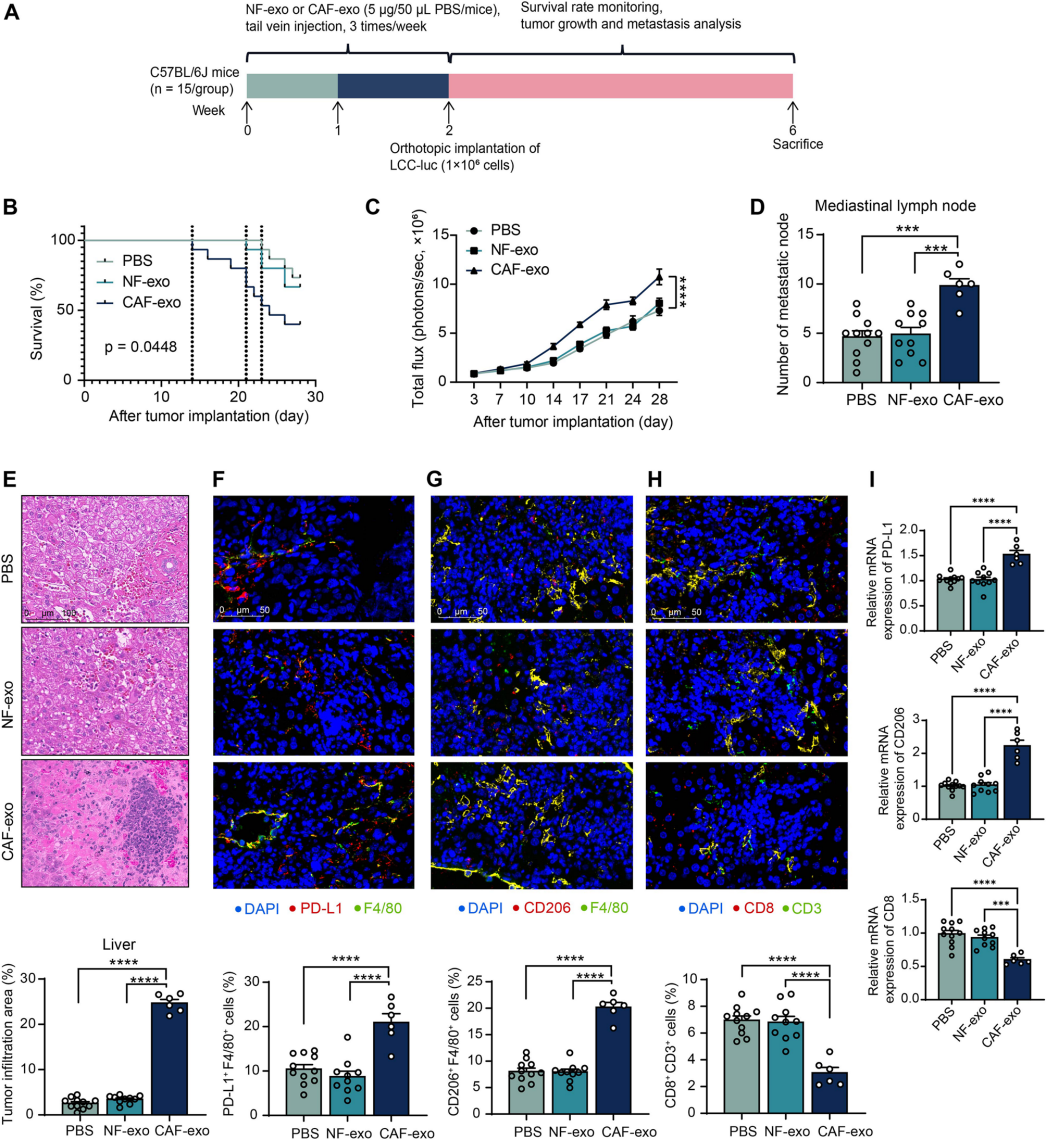
CAF-exo enhance the growth and distant metastasis of LLC in mice. (**A**) Schematic for experimental design. (**B**) The survival of mice over 4 weeks was analyzed using the log-rank test. (**C**) Bioluminescence imaging to monitor tumor growth and metastasis. (**D**) Tumor lymph node metastasis in mice after euthanasia. (**E**) The tumor infiltration area in liver tissues was observed using HE staining. Immunofluorescence detection of PD-L1 (**F**) and CD206 (**G**) expression in liver infiltrating tumor tissues. (**H**) Immunofluorescence detection of CD3^+^CD8^+^ T cell percentage in liver infiltrating tumor tissues. (**I**) Expression of CD8, PD-L1, and CD206 in liver tissues of mice after CAF-exo and NF-exo injections by RT-qPCR. *N* = 6–15 (The number of mice differs in each group due to changes in survival). The data are shown as mean ± SEM. ***p < 0.001, ****p < 0.001 (ANOVA)

The immunosuppressive phenotype of macrophages induced by CAF-exo has been confirmed above. However, whether it can play the same role in vivo remains to be investigated. Mice were pretreated with PBS, CAF-exo, or NF-exo through the tail vein, followed by orthotopic implantation of luciferase-labeled mouse LLC (LLC-Luc) cells (Fig. 3A). The survival of mice was monitored (Fig. 3B). The first dead mouse appeared on day 23 in the PBS group, NF-exo on day 21, and CAF-exo on day 14. The difference in OS between the PBS and NF-exo groups of mice was insignificant, whereas survival was significantly lower in mice pretreated with CAF-exo than in mice pretreated with PBS or NF-exo. Bioluminescence imaging also showed that mice pretreated with CAF-exo had enhanced tumor colonization, and the presence of distant metastatic tumor foci was observed in the CAF-exo-treated mice in the second week. By contrast, the remaining two groups of mice began to show visible liver metastases only in the fourth week (Fig. 3C).

To confirm the formation of PMN and the presence of distant metastases, the presence of pulmonary mediastinal lymph node metastases was observed after the euthanasia of the remaining surviving mice. The mediastinal lymph node metastases were increased in the CAF-exo group of mice (Fig. 3D). Liver metastases were observed using HE staining. Only a small number of tumor metastatic foci were observed in the PBS or NF-exo group of mice, whereas liver infiltration of the tumors was significantly increased in CAF-exo-treated mice (Fig. 3E). Immunofluorescence staining and RT-qPCR observed an increase in PD-L1- and CD206-expressing macrophages (F4/80^+^) and a decrease in CD3^+^CD8^+^ T cells (Fig. 3F-I) in liver-infiltrating tumor tissues of mice in the CAF-exo group.

### CAF-exo encapsulate GREM1 essential for macrophage M2 polarization

To investigate the specific molecular mechanisms by which CAF-exo induce macrophage immunosuppressive phenotypic switching and PMN formation, we first focused on the expression of specific genes in CAF. CAF (GSM564890-GSM564904) and NF (GSM564905-GSM564919) samples from the GEO dataset GSE22862 (https://www.ncbi.nlm.nih.gov/geo/query/acc.cgihttps://www.ncbi.nlm.nih.gov/geo/query/acc.cgi? acc=GSE22862) were used to filter differentially expressed genes using the R language limma package (https://rdrr.io/bioc/limma/). Sixty differentially expressed gene IDs (Fig S3A) in CAF were screened using *p* < 0.05, |logFC| >1 as the significance cutoff threshold. The IDs were annotated by the GPL5175 platform (https://www.ncbi.nlm.nih.gov/geo/query/acc.cgi? acc=GPL5175) and the R software package clusterProfiler (https://rdrr.io/bioc/clusterProfiler/) for GO and KEGG enrichment analysis to functionally characterize the differentially expressed genes. In the GO: molecular function (MF) enrichment analysis, we observed that the sixty genes could be enriched for cytokine activity, receptor-ligand activity, and signaling receptor activator activity that are closely related to intercellular signaling regulation (Fig S3B). KEGG analysis showed that cytokine-cytokine receptor interaction was the pathway with the highest enrichment of differentially expressed genes in CAF (Fig S3C). Therefore, we constructed the protein-protein interaction network of the genes enriched in the relevant pathways by STRING (https://version-12-0.string-db.org/) and observed the presence of four central proteins: FGF10, CXCL12, BMP4, and GREM1 (Fig S3D). CAF-derived CXCL12 has been reported to mediate macrophage M2 polarization to promote NSCLC progression (Wu et al. 2022). Therefore, BMP4, GREM1, and FGF10, which have not been reported, were selected for further study. The correlation between the expression of BMP4, GREM1, and FGF10 and the expression of macrophage M2 polarization markers CD163 and CD206 in NSCLC tissues was analyzed by GEPIA (http://gepia.cancer-pku.cn/index.html) (Fig S3E). Only GREM1 was significantly positively correlated with the CD163 and CD206 (MRC1) expression.

**Fig. 4:**
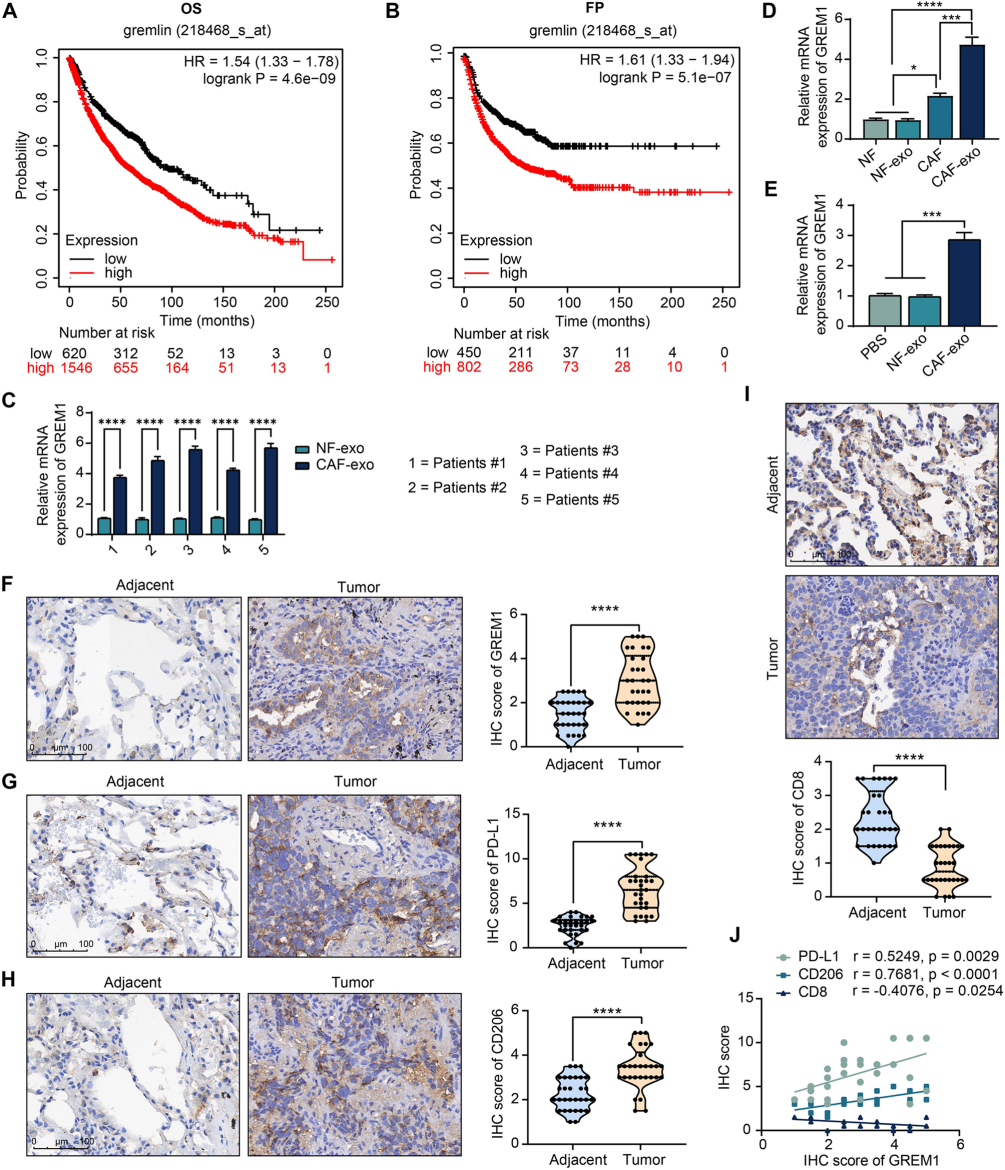
CAF-exo encapsulate GREM1. Kaplan-Meier Plotter database analysis of the prognostic significance of GREM1 expression in NSCLC patients on OS (**A**) and FP (**B**). (**C**) The mRNA expression of GREM1 in NF-exo and CAF-exo from each NSCLC patient was examined using RT-qPCR. (**D**) The mRNA expression of GREM1 in fusion samples from NSCLC patients was examined using RT-qPCR. (**E**) The mRNA expression of GREM1 in macrophages after treatment with PBS, NF-exo, or CAF-exo was examined using RT-qPCR. Immunohistochemical detection of GREM1 (**F**), PD-L1 (**G**), CD206 (**H**), and CD8 (**I**) expression in tumor tissues and adjacent tissues of NSCLC patients (*N* = 30). (**J**) Correlation between GREM1 expression and PD-L1, CD206, CD8 expression in NSCLC tissues (*N* = 30). The results are representative of data from triplicate experiments. The data are shown as mean ± SEM. *p < 0.05, ***p < 0.001, ****p < 0.001 (t-test or ANOVA)

The Kaplan-Meier Plotter (https://kmplot.com/analysis/index.php? p=service&cancer=lung) database showed that GREM1 expression in NSCLC patients predicted worse OS and first progression (FP) (Fig. 4A, B). However, whether CAF-exo depend on GREM1 to regulate PMN formation is not yet known. The expression of GREM1 in CAF-exo and NF-exo from each NSCLC patient was examined, and significantly elevated expression of GREM1 was observed in CAF-exo (Fig. 4C). Significantly enhanced expression of GREM1 in CAF compared to NF and was further enriched in CAF-exo, as revealed by RT-qPCR (Fig. 4D). GREM1 expression was not significantly changed in macrophages after NF-exo treatment, whereas it was significantly increased in macrophages after CAF-exo treatment (Fig. 4E).

The expression of GREM1, PD-L1, CD206, and CD8 was detected in paraffin-embedded tumor tissues and adjacent tissues of NSCLC patients (*n* = 30), and the enhancement of the expression of GREM1, PD-L1, and CD206, as well as the attenuation of the expression of CD8 were observed in tumor tissues (Fig. 4F-I). Moreover, the expression of GREM1 in tumor tissues was significantly and positively correlated with the PD-L1 and CD206 expression (Fig. 4J).

### CAF-exo affect macrophage immunosuppressive phenotype switching via GREM1

To analyze the role of GREM1 encapsulated by CAF-exo, we first transfected two shRNA sequences of GREM1 into CAF to knock down its expression (a nonsense sequence as a control). RT-qPCR and western blot analysis confirmed the knockdown efficiency (Fig S4A). The exosomes generated from CAF after different treatments were collected and fused isometrically to obtain Exo-scramble, Exo-KD 1#, and Exo-KD 2#. NTA and TEM analyses revealed that the transfection of shRNA did not lead to a change in the quality of exosomes produced by CAF, and the exosomes remained elliptical with a diameter of about 80–120 nm (Fig S4B, C). All exosomes expressed CD63, CD81, and TSG101 but not Calnexin (Fig S4D). The knockdown of GREM1 expression in CAF was followed by a reduction in the expression of GREM1 in its derived exosomes (Fig S4E). Human macrophages (differentiated from monocytes of human peripheral blood samples) were confirmed to take up these three exosomes by PKH67 labeling (Fig S4F).

**Fig. 5:**
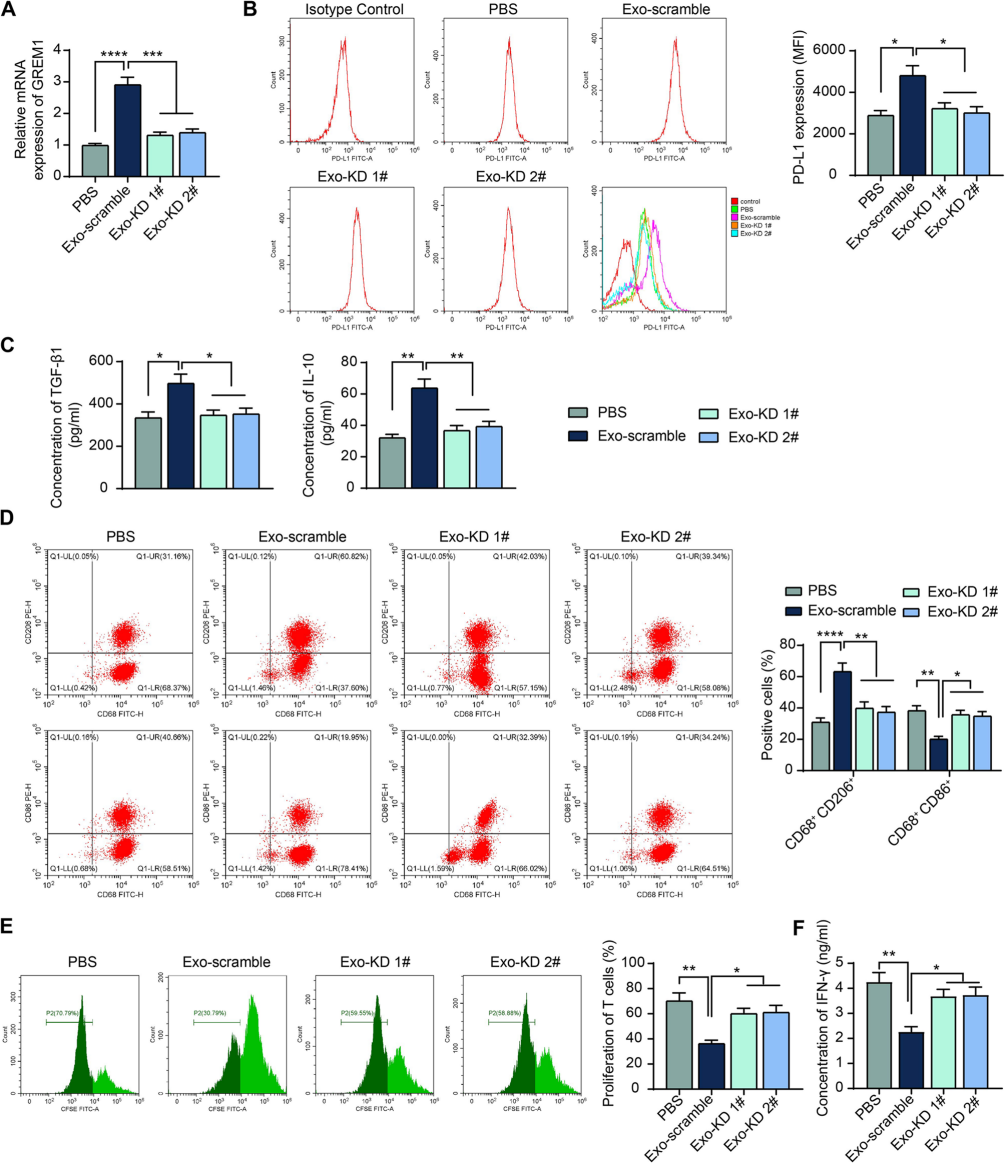
Knockdown of GREM1 inhibits CAF-exo-mediated macrophage M2 polarization. CAF were transfected with two different shRNA sequences or nonsense sequences (Scramble), and the derived exosomes were extracted (named Exo-KD 1#, Exo-KD 2#, and Exo-Scramble). (**A**) GREM1 mRNA expression in macrophages treated with exo derived from CAF with different transfections or PBS was analyzed using RT-qPCR. (**B**) The surface expression of PD-L1 in macrophages was analyzed by flow cytometry. (**C**) Immunosuppressive factors TGF-β1 and IL-10 released by macrophages were analyzed using ELISA. (**D**) M1/M2 polarization of macrophages was analyzed using flow cytometry. (**E**) The proliferation of T cells co-cultured with macrophages was analyzed using flow cytometry. (**F**) The effect of co-culture with macrophages on IFN-γ release from T cells was analyzed using ELISA. The results are representative of data from triplicate experiments. The data are shown as mean ± SEM. *p < 0.05, **p < 0.01, ***p < 0.001, ****p < 0.001 (ANOVA)

Compared to the Exo-scramble, the ability of Exo-KD 1# or Exo-KD 2# to promote GREM1 expression in macrophages was significantly attenuated (Fig. 5A). After knocking down the expression of GREM1, the promotion effects of CAF-exo on macrophage PD-L1 expression and TGF-β1 and IL-10 release were significantly weakened (Fig. 5B, C). Knockdown of GREM1 resulted in a blockade of the M2 phenotype polarization (Fig. 5D). The proliferation of T cells inhibited by macrophages was significantly improved (Fig. 5E), and the release of killer factors from T cells was activated in response to GREM1 knockdown (Fig. 5F).

### Knockdown of GREM1 inhibits distant metastasis induced by CAF-exo

**Fig. 6:**
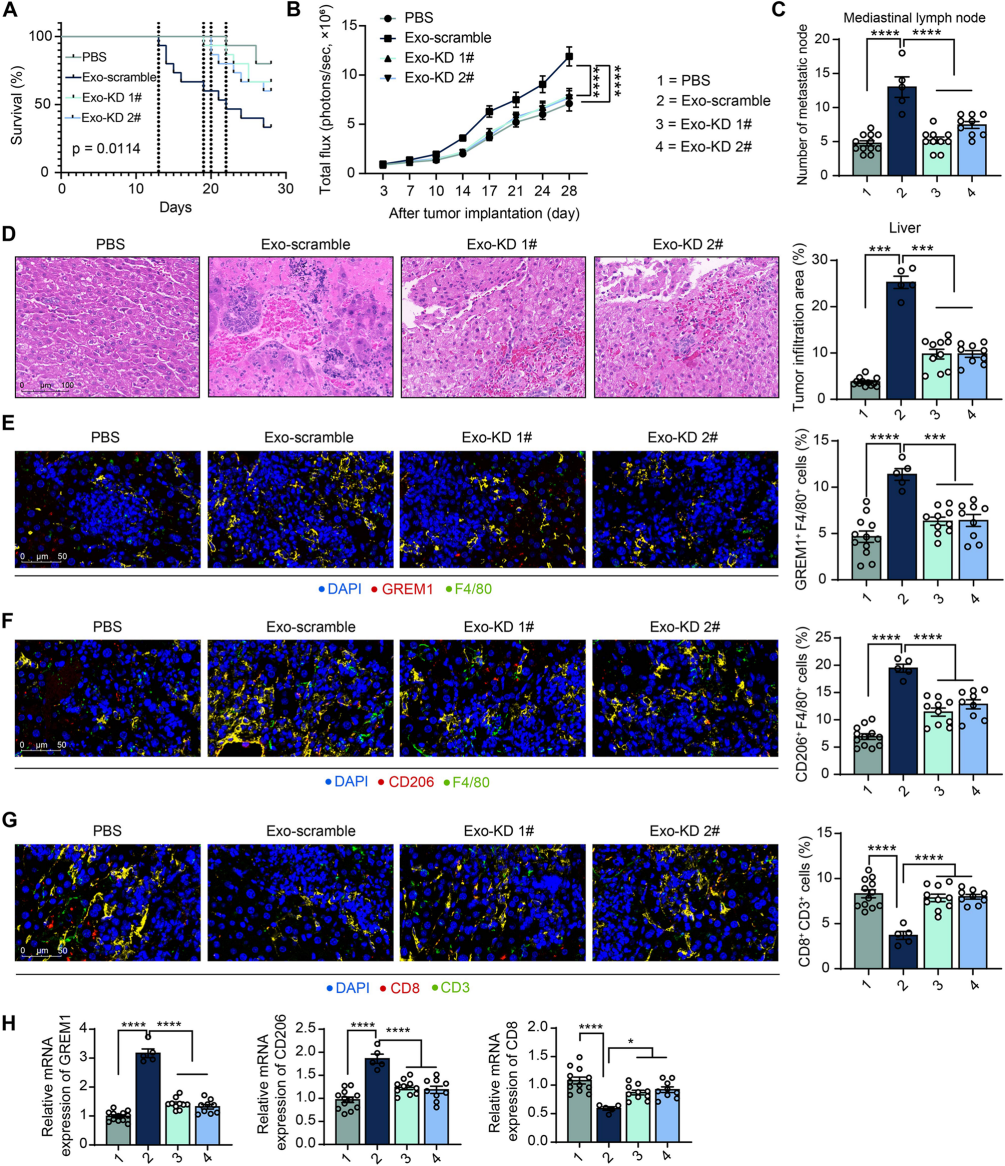
Knockdown of GREM1 inhibits CAF-exo-mediated distant metastasis. (**A**) The survival of mice over 4 weeks was analyzed using the log-rank test. (**B**) Bioluminescence imaging to monitor tumor growth. (**C**) Tumor lymph node metastasis in mice after euthanasia. (**D**) The tumor infiltration area in liver tissues was observed using HE staining. Immunofluorescence detection of GREM1^+^F4/80^+^ (**E**), CD206^+^F4/80^+^ (**F**), and CD8^+^CD3^+^ (**G**) cell population in liver infiltrating tumor tissues. (**H**) Expression of GREM1, CD206, and CD8 in liver-infiltrating metastatic tumor tissues by RT-qPCR. *N* = 5–15 (The number of mice differs in each group due to changes in survival). The data are shown as mean ± SEM. *p < 0.05, ***p < 0.001, ****p < 0.001 (ANOVA)

LLC mice were also treated with exosomes derived from CAF after transfection according to the protocol described previously. Knockdown of GREM1 in CAF-exo significantly improved survival and delayed death in tumor-bearing mice (Fig. 6A). Bioluminescence imaging documented that the knockdown of GREM1 in CAF-exo significantly reduced tumor growth (Fig. 6B).

The remaining surviving mice were euthanized, mice injected with Exo-KD 1# and Exo-KD 2# showed a significant decline in lymph node metastasis of the tumors (Fig. 6C) and reduced infiltration of cancer cells into the liver relative to mice injected with Exo-scramble (Fig. 6D). GREM1 expression in macrophages (F4/80^+^) of liver infiltrating tumor tissues was enhanced in Exo-scramble-treated mice compared with PBS-treated mice, and GREM1 expression in macrophages of liver metastases was significantly reduced in mice pre-treated with Exo-KD 1# and Exo-KD 2# (Fig. 6E). Knockdown of GREM1 expression in CAF-exo significantly reduced M2-polarized macrophages (CD206^+^F4/80^+^) and enhanced the infiltration of CD8^+^CD3^+^ T cells in liver-infiltrating tumor tissues (Fig. 6F, G). As revealed by RT-qPCR, CAF-exo treatment enhanced expression of GREM1 and CD206 as well as reduced expression of CD8 in liver-infiltrating tumor tissues, whereas knocking down the expression of GREM1 in exosomes significantly attenuated CAF-exo-induced gene expression changes (Fig. 6H).

### GREM1 maintains a FGF4/SHH positive feedback loop to promote M2 polarization in macrophages

**Fig. 7:**
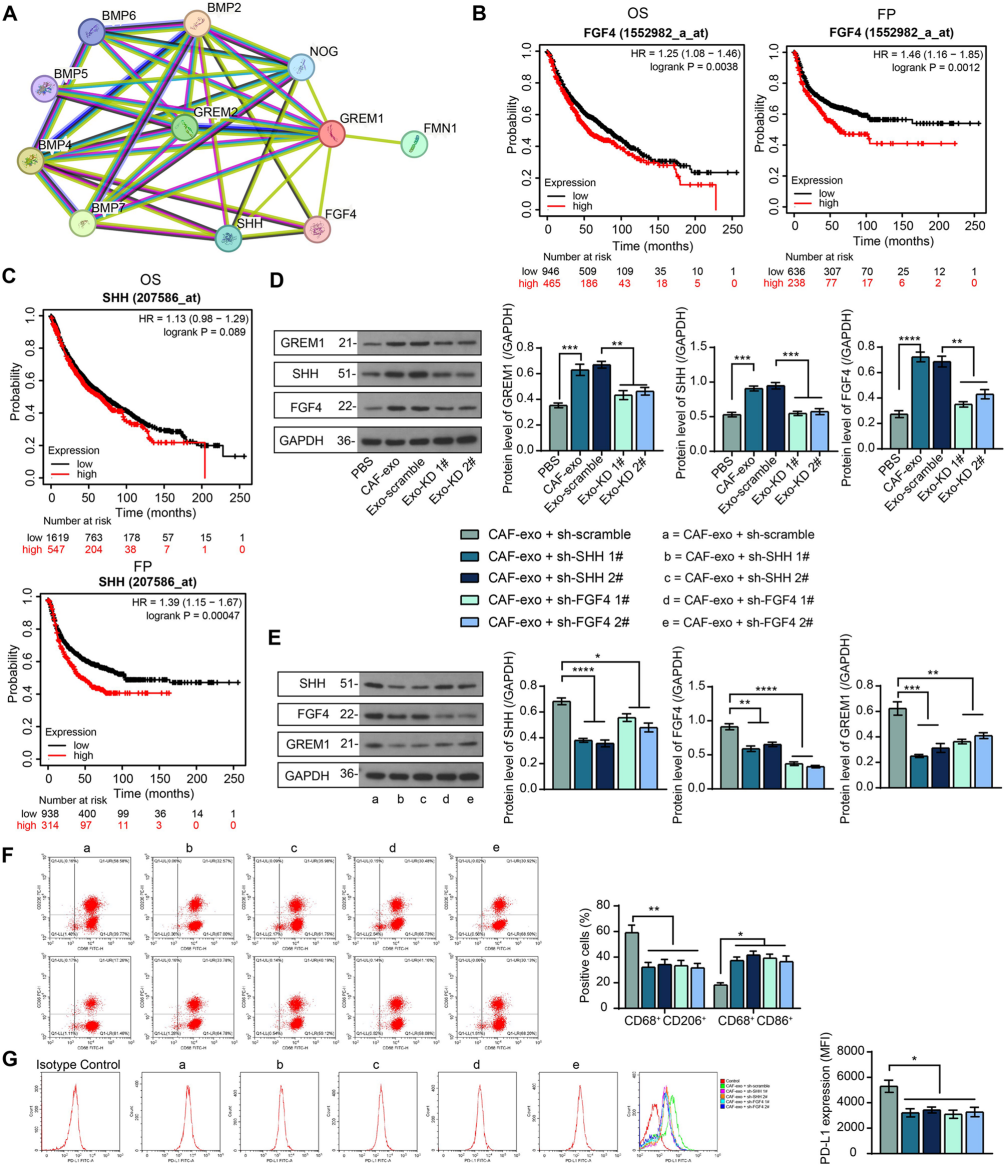
Blockade of FGF4/SHH signaling in macrophages inhibits GREM1-mediated macrophage phenotype switching. (**A**) The protein-protein interaction network of GREM1 was constructed in STRING. Prognostic significance of FGF4 (**B**) and SHH (**C**) expression on OS and FP in NSCLC patients. (**D**) The effect of exo derived from CAF with different transfection or PBS on GREM1, SHH, and FGF4 protein expression in macrophages was analyzed using western blot. Macrophages after CAF-exo treatment were transfected with shRNAs targeting SHH 1#, 2#, FGF4 1#, FGF4 2#, or sh-scramble. (**E**) The protein expression of GREM1, SHH, and FGF4 in macrophages was analyzed using western blot. (**F**) The effect of blocking FGF4/SHH expression on macrophage polarization was analyzed using flow cytometry. (**G**) Changes in PD-L1 expression on the surface of macrophages were analyzed by flow cytometry. The results are representative of data from triplicate experiments. The data are shown as mean ± SEM. *p < 0.05, **p < 0.01, ***p < 0.001, ****p < 0.001 (ANOVA)

We confirmed that CAF-exo induced macrophage M2 polarization by delivering GREM1, but the downstream molecular signaling of GREM1 in macrophages remains to be revealed. We analyzed the proteins with potential interaction with GREM1 in STRING, setting high confidence (0.7) (Fig. 7A). We observed that GREM1 interacts with BMPs and SHH, and FGF4. Kaplan-Meier Plotter analysis showed that FGF4 expression predicted worse OS and FP in patients (Fig. 7B). Even though the prognostic significance of SHH on OS was not significant, it predicted significantly worse FP in patients (Fig. 7C).

Enhanced SHH and FGF4 protein expression was observed in CAF-exo-treated macrophages (differentiated from monocytes in human peripheral blood), whereas their expression in macrophages was significantly weakened by knocking down the level of GREM1 in CAF-exo (Fig. 7D). To reveal whether FGF4/SHH is responsible for the macrophage immunophenotypic switch promoted by GREM1 in CAF-exo, we transfected shRNAs for SHH or FGF4 into CAF-exo-treated macrophages. shRNAs targeting SHH or FGF4 not only interfered with FGF4/SHH expression but also led to a decrease in GREM1 expression, demonstrating the existence of a feedback loop (Fig. 7E). Flow cytometric detection of blockade of the FGF4/SHH/GREM1 feedback loop resulted in a phenotypic switch to M1-phenotype (CD68^+^CD86^+^) macrophages and a reduction in the proportion of M2-phenotype (CD68^+^CD206^+^) macrophages (Fig. 7F). Consistently, a significant reduction in the surface expression of PD-L1 was observed (Fig. 7G).

GREM1 knockout (KO) macrophages were constructed, and western blot analysis showed the absence of endogenous GREM1 expression in the knockout cells (Fig S5A). Exogenous overexpression (OE) plasmids of GREM1/FGF4/SHH were transfected into GREM1-KO cells, respectively. Western blot analysis (Fig S5B) observed successful overexpression of FGF4/SHH, as well as successful exogenous expression of GREM1. It is worth stating that FGF4/SHH was unable to induce GREM1 expression in GREM1-KO cells. When GREM1 is not expressed, SHH is unable to significantly activate FGF4 expression, and SHH requires GREM1 to form a positive feedback loop (GREM1/FGF4/SHH) to affect its upstream FGF4. Flow cytometry (Fig S5C) detected that FGF4/SHH expression was not effective in influencing macrophage polarization when GREM1 was not present. In the presence of exogenous GREM1, FGF4/SHH expression effectively promoted M2 polarization and inhibited M1 polarization in macrophages. Macrophage surface PD-L1 expression also followed the same trend as M2 polarization (Fig S5D). This suggests that the pro-macrophage immunosuppressive phenotype of FGF4/SHH is dependent on GREM1 expression.

### Positive feedback regulation of FGF4/SHH and GREM1 promotes distant tumor metastasis in LLC mice

**Fig. 8:**
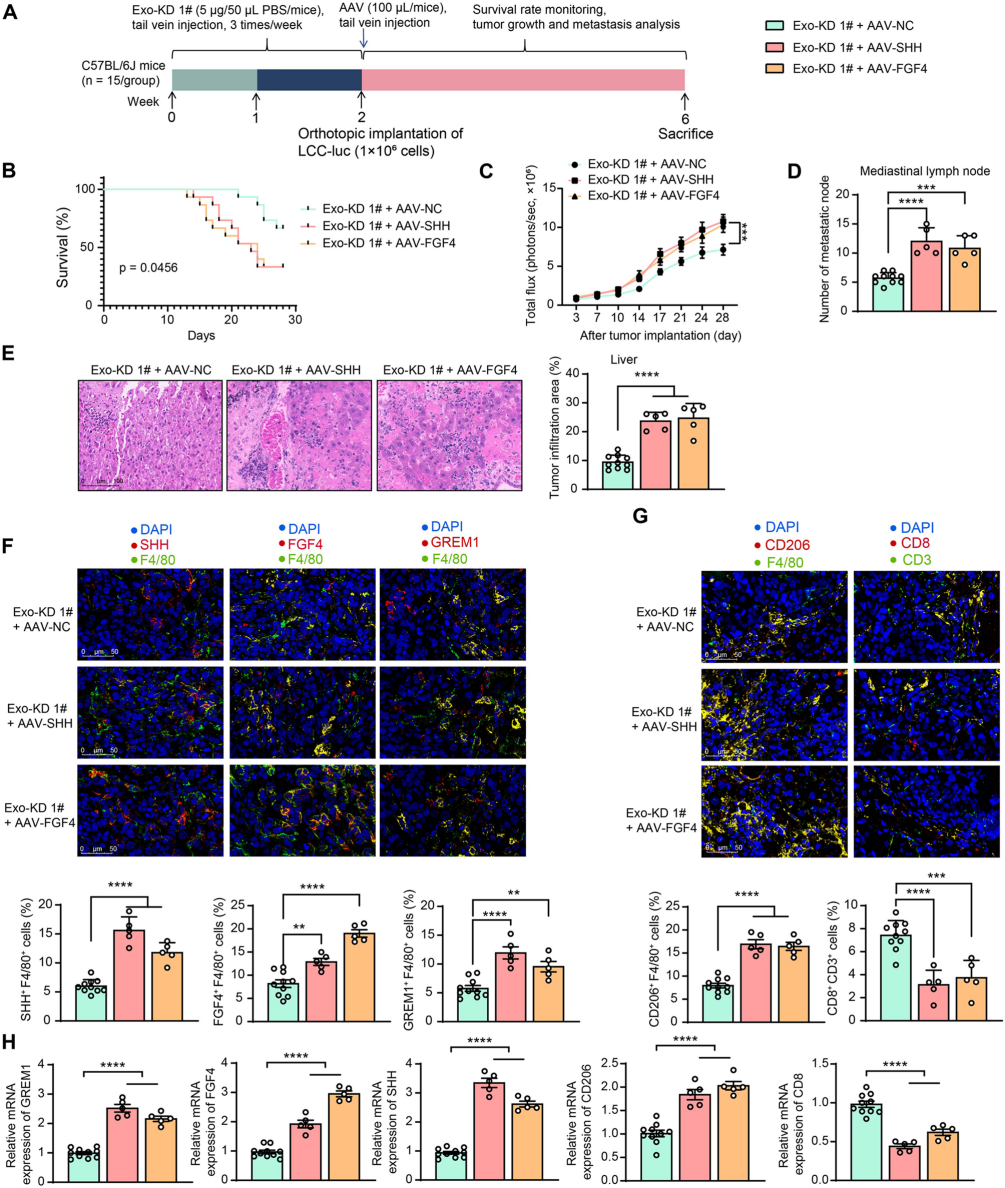
Overexpression of SHH or FGF4 potentiates tumor colonization and distant metastasis in the presence of GREM1 knockdown. (**A**) Schematic for experimental design. (**B**) The survival of mice over 4 weeks was analyzed using the log-rank test. (**C**) Bioluminescence imaging to monitor tumor growth. (**D**) Tumor lymph node metastasis in mice after euthanasia. (**E**) The tumor infiltration area in liver tissues was observed using HE staining. (**F**) Immunofluorescence detection of GREM1, FGF4, and SHH expression in macrophages (F4/80^+^) liver infiltrating tumor tissues. (**G**) Immunofluorescence detection of CD206^+^F4/80^+^ macrophage and CD8^+^CD3^+^ T cell population in liver infiltrating tumor tissues. (**H**) The mRNA expression of FGF4, SHH, GREM1, CD206, and CD8 in liver infiltrating tumor tissues was examined using RT-qPCR. *N* = 5–15 (The number of mice differs in each group due to changes in survival). The data are shown as mean ± SEM. **p < 0.01, ***p < 0.001, ****p < 0.001 (ANOVA)

To investigate whether FGF4/SHH could support tumor progression inhibited by knockdown of GREM1, we pre-treated CAF-derived exosomes (Exo-KD 1#) in combination with AAV carrying an F4/80 promoter of SHH (Exo-KD 1# + AAV-SHH) or FGF4 (Exo-KD 1# + AAV-FGF4) or control AAV-NC (Exo-KD 1# + AAV-NC), followed by LLC administration (Fig. 8A). The injection of either AAV-SHH or AAV-FGF4 shortened the survival of tumor-bearing mice (Fig. 8B) and promoted tumor growth (Fig. 8C). The number of metastatic nodes in the mediastinal lymph nodes of the lungs was significantly enhanced in AAV-SHH- or AAV-FGF4-treated mice (Fig. 8D), and the infiltrating area of metastatic tumors in the liver tissue was significantly increased (Fig. 8E). Immunofluorescence in liver-infiltrating tumor tissues showed that AAV-SHH or AAV-FGF4 successfully activated the positive feedback regulation between FGF4/SHH and GREM1 in macrophages (F4/80^+^) (Fig. 8F), which contributed to an increase in CD206^+^F4/80^+^ macrophages and a decline in CD8^+^CD3^+^ T cells (Fig. 8G). RT-qPCR reconfirmed positive feedback activation of FGF4/SHH/GREM1, as well as increased expression of CD206 and decreased expression of CD8 in liver tissues (Fig. 8H).

## Discussion

**Fig. 9:**
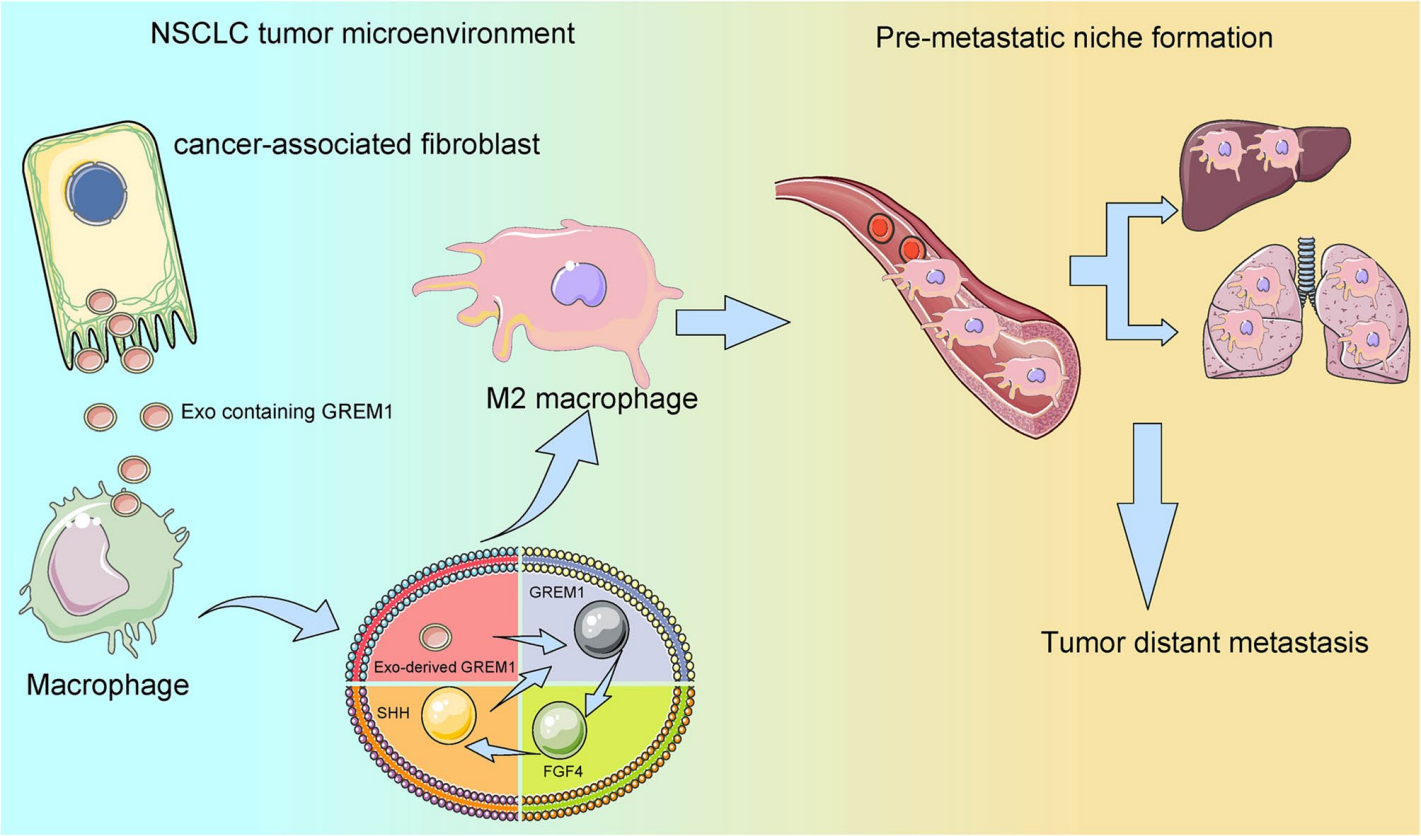
Schema depicting CAF-exo-induced PMN formation via encapsulating GREM1 and maintaining a positive feedback loop between GREM1 and FGF4/SHH

The biology of extracellular vesicles has been implicated in many malignant processes, including inflammatory responses, epithelial-to-mesenchymal transition, and the formation of PMN (Saviana et al. 2021). Furthermore, CAFs have been recently speculated to exert important functions in epithelial solid tumors, such as tumor growth and metastasis, via the secretion of factors that promote tumorigenesis by stimulating cancer cell proliferation and invasion, in addition to recruiting macrophages and suppressing T-cell antitumor immunity (Shintani et al. 2023). In the study here, we verified that CAF-exo promoted the formation of PMN via polarizing macrophages toward an immunosuppressive phenotype. CAF-exo encapsulated GREM1 and consequently activated the FGF4/SHH signaling in macrophages (Fig. 9).

CAF-extracellular vesicles induced lung pre-metastatic niche formation in mice and increased salivary adenoid cystic carcinoma lung metastasis, with the main focus on activation of fibroblasts (Kong et al. 2019). Interestingly, Morrissey et al. revealed that tumor-derived exo polarized macrophages towards an immunosuppressive phenotype characterized by upregulating PD-L1 (Morrissey et al. 2021). TGF-β1 has been shown to inhibit the activation of T cells and the expression of PD-L1 by decreasing the expression of IFN-γ and granzyme B in T cells (Pu and Ji 2022). Therefore, we attributed the promotion of PMN markers FN, LOX, and MMP9 in the liver and lung tissues of mice injected with CAF-exo to immunosuppressive macrophages since the PD-L1 and CD206 expression was enhanced in these tissues. Moreover, the immunosuppressive macrophages induced by CAF-exo in vitro also repressed T cell proliferation and IFN-γ levels, primarily attesting to our hypothesis. Mice with lung tumors and liver metastasis induced by CAF-exo also exhibited shortened survival and higher PD-L1 and CD206 levels and lower CD8 levels in the liver than mice injected with NF-exo and PBS, indicating immunosuppressive macrophages and impaired T cell function.

As for the payloads of CAF-exo, long noncoding RNAs have been widely explored in different cancers (Sun et al. 2023; Yang et al. 2022a). With available databases, we identified GREM1 as a possible cargo of CAF-exo that correlated with both macrophage markers CD163 and CD206. Davis et al. demonstrated that fibroblasts^GREM1+^ positively correlated with total macrophages (CD68^+^) and M2 macrophages (CD163^+^) in pancreatic ductal adenocarcinoma (Davis et al. 2022). Mthunzi et al. showed that recombinant GREM1 potentiated M2-like polarization of mouse macrophages, whereas genetic depletion of GREM1 in bone marrow-derived macrophages inhibited M2 polarization (Mthunzi et al. 2023). GREM1-positive NSCLC patients had much higher levels of CD3^+^ T and sharply lower levels of CD8^+^ T than GREM1-negative patients (Bao et al. 2023), suggesting its expression was related to immune cell infiltration. To further deepen our study, two shRNA sequences were used to transfect CAF and extract exosomes. It was observed that the M2 polarization of macrophages induced by CAF-exo and the impaired T cell function were blocked by GREM1 knockdown in CAF. Fregni et al. revealed that the reconstitution of normal adjacent lung tissue-derived mesenchymal stem cells with four genes, GREM1, LOXL2, ADAMTS12, and ITGA11, contributed to the increased ability of tumor-associated mesenchymal stem cells to induce primary tumor cell dissemination (Fregni et al. 2018), indicating its possible linkage to PMN formation. More relevantly, GREM1 promoted NSCLC cell migration and invasion, whereas shGREM1 had the opposite effect (Kan et al. 2022). Our in vivo evidence also implicated that the knockdown of GREM1 was beneficial for survival and less metastasis in mice.

GREM1 has been established to exert a potent inhibitory action via binding to and forming heterodimers with BMP-2, BMP-4, and BMP-7 (Wordinger et al. 2008), while the positive feedback loop of GREM1 and the SHH/FGF4 signaling has only been validated in the developing mouse limb bud (Farin et al. 2013). Petty et al. showed that tumor-derived SHH drives M2 polarization, thereby suppressing CD8^+^ T cell recruitment (Petty et al. 2019). FGF4 is one of the representative paracrine FGFs binding to heparan-sulfate proteoglycan and fibroblast growth factor receptors, which are frequently amplified and overexpressed in lung cancer (Katoh 2016). In the present study, the M2 polarization in response to CAF-exo was found to be reversed by the knockdown of FGF4 or SHH, which occurred concomitantly with diminished GREM1 in macrophages, indicating that the maintenance of this feedback loop was at least partially responsible for the immunosuppressive role of CAF-exo on macrophages. The in vivo evidence further confirmed that the AVV-FGF4 or AAV-SHH induced the formation of PMN in LLC mice in the presence of Exo-KD 1#.

Further studies are needed to expound on how the CAFs become activated in the niche before the macrophages. In addition, whether the regulation of CAF-exo in PMN formation and distant metastasis through the GREM1/SHH/FGF4 axis can similarly influence orthotopic tumor properties to promote the distant metastasis needs to be further investigated.

In summary, our study showed that GREM1 encapsulated by CAF-exo promotes the formation of PMN in NSCLC by remodeling the macrophage immunosuppressive phenotype, which results from the FGF4/SHH feedback loop. From a therapeutic perspective, this study provides a rationale for the concept of therapeutic targeting of CAF in NSCLC.

## Supplementary Information

Supplementary Material 1.

## Data Availability

No datasets were generated or analysed during the current study.
