# Supplementary material for: CAF-derived exosomes drive the FGF4/SHH feedback loop by encapsulating GREM1 in non-small cell lung cancer
Source: Mol Med. 2025 Aug 23;31:281. doi: 10.1186/s10020-025-01340-0 (PMC12375272; doi:10.1186/s10020-025-01340-0)
Supplement: Supplementary file 1 — Supplementary Material 1. [file 10020_2025_1340_MOESM1_ESM.docx]

**Supplementary Figure 1**

**
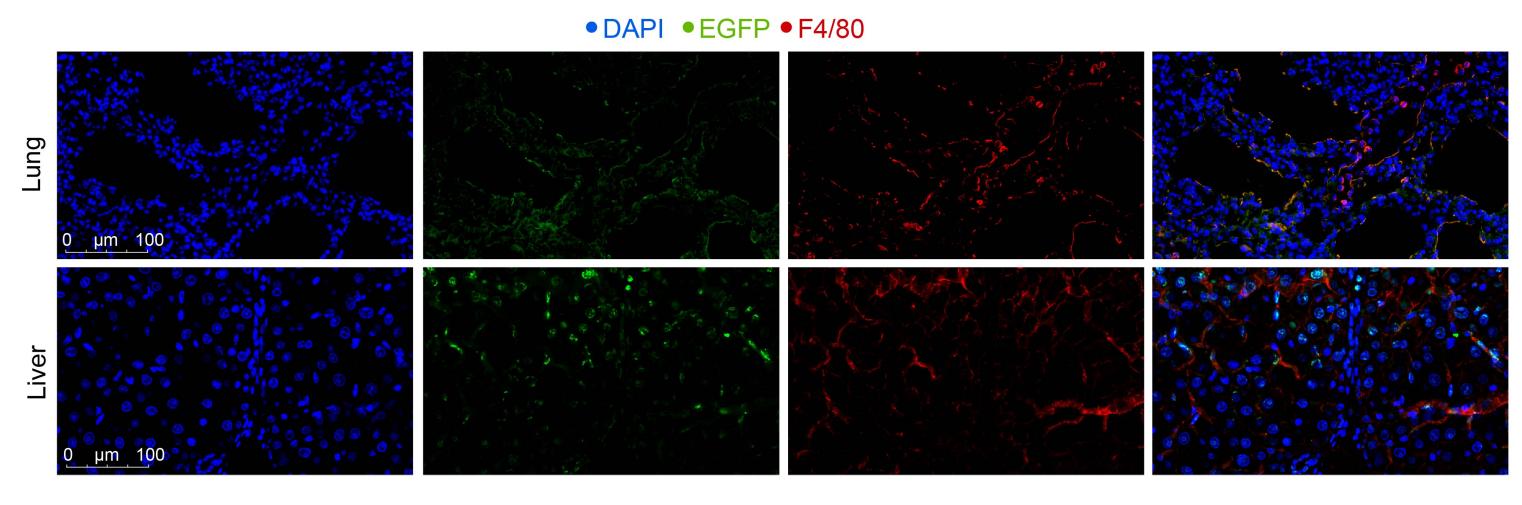
**

**Supplementary Figure 1** The infection efficiency on macrophages (F4/80^+^) in lung and liver tissues after two weeks of EGFP-labeled AAV vectors.

**Supplementary Figure 2**


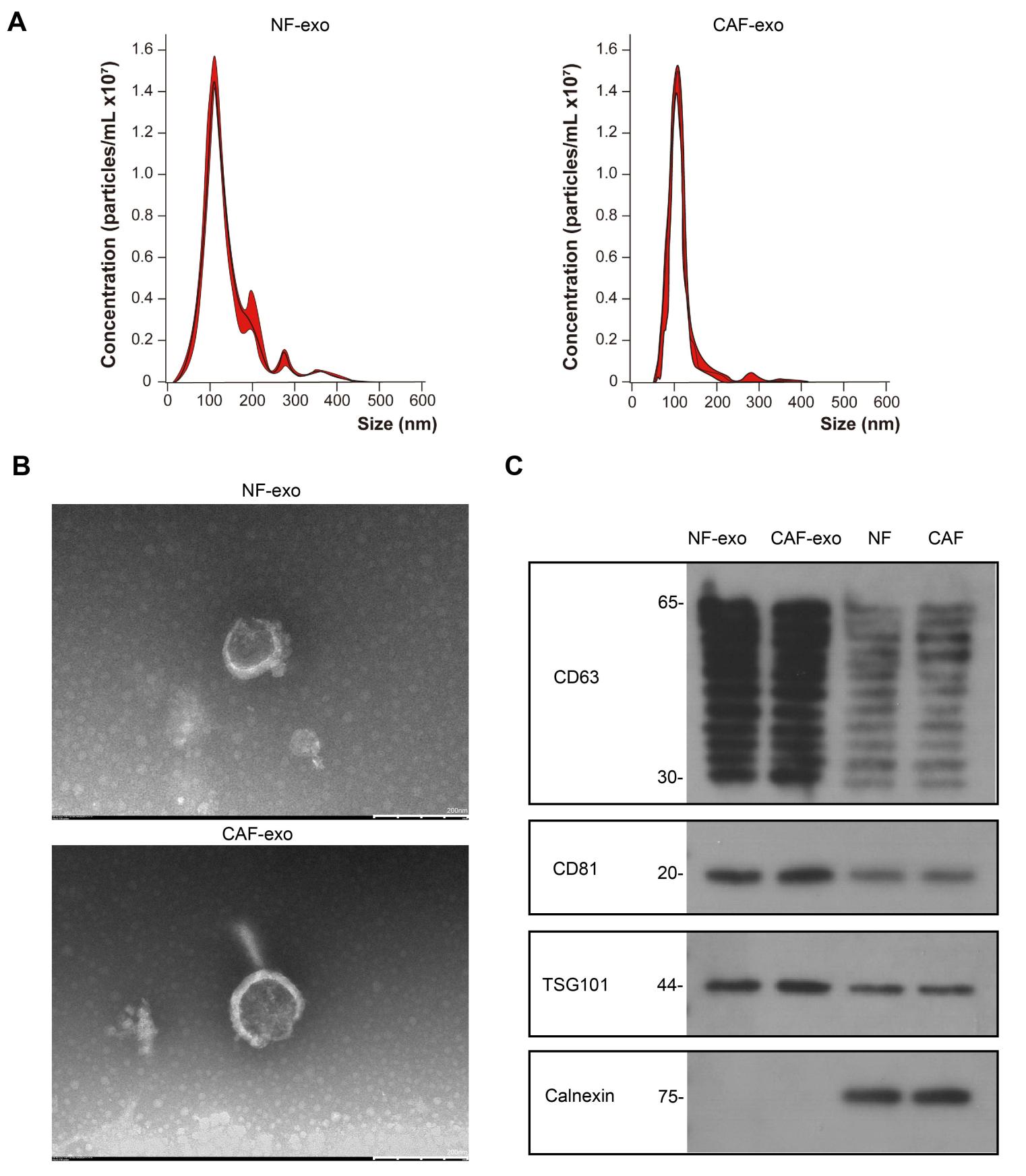


**Supplementary Figure 2** Characterization of CAF-exo and NF-exo. (A) The particle size of exosomes obtained from NF and CAF was examined using NTA. (B) Morphologic structure of exosomes observed by TEM. (C) Expression of marker proteins for exosomes in CAF, CAF-exo, NF, and NF-exo was detected by western blot.

**Supplementary Figure 3**

**
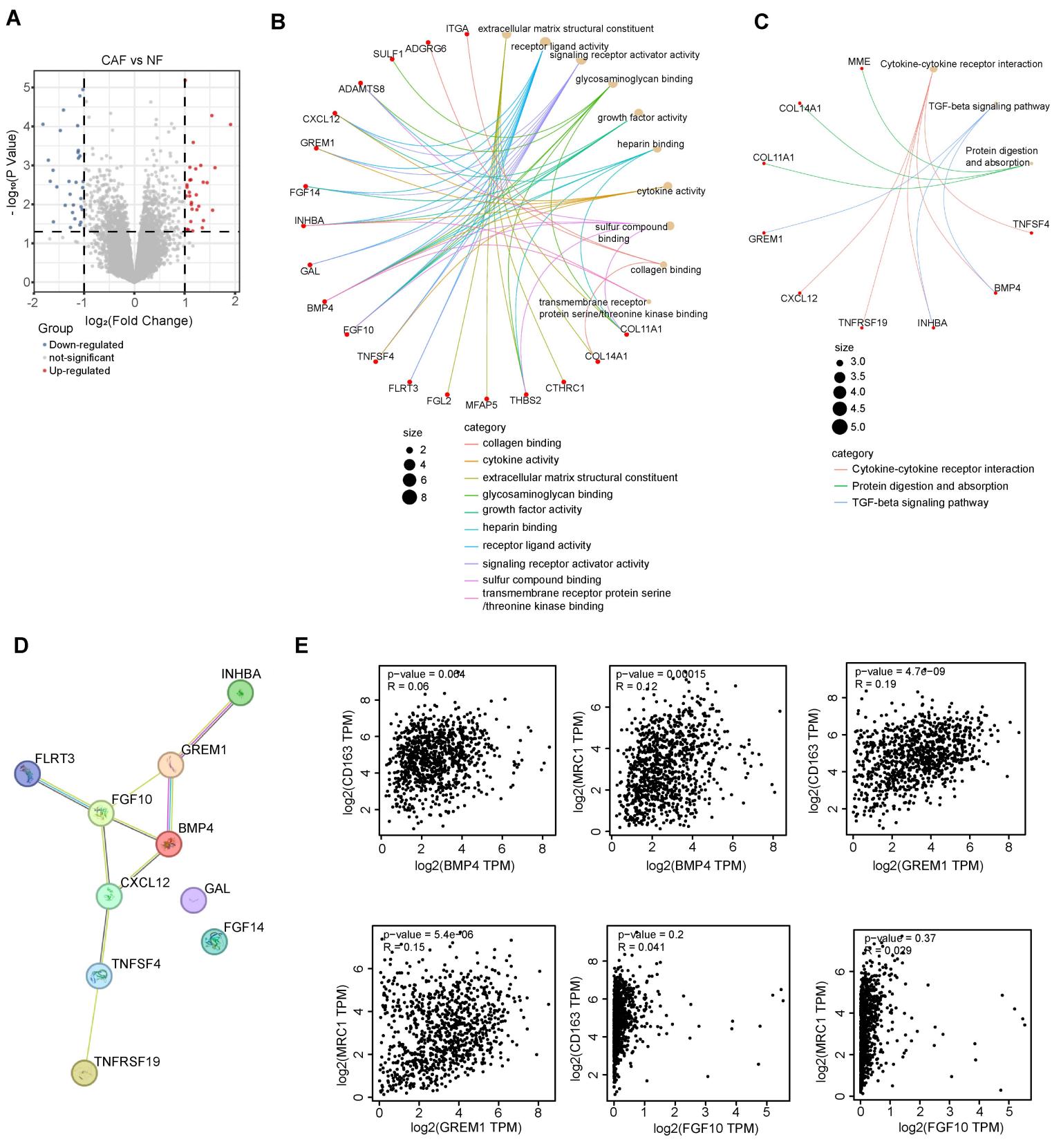
**

**Supplementary Figure 3** GREM1 is encapsulated by CAF-exo. (A) Genes differentially expressed in CAF *vs* NF in the GSE22862 dataset. (B) GO: MF enrichment analysis plot and KEGG pathway enrichment analysis plot (C) of differentially expressed genes. (D) Interaction analysis of proteins enriched in relevant pathways at the String database. (E) The correlation between the expression of FGF10, BMP4, GREM1, and the expression of M2 polarization markers CD163 and CD206 in NSCLC was analyzed in the GEPIA database.

**Supplementary Figure 4**

**
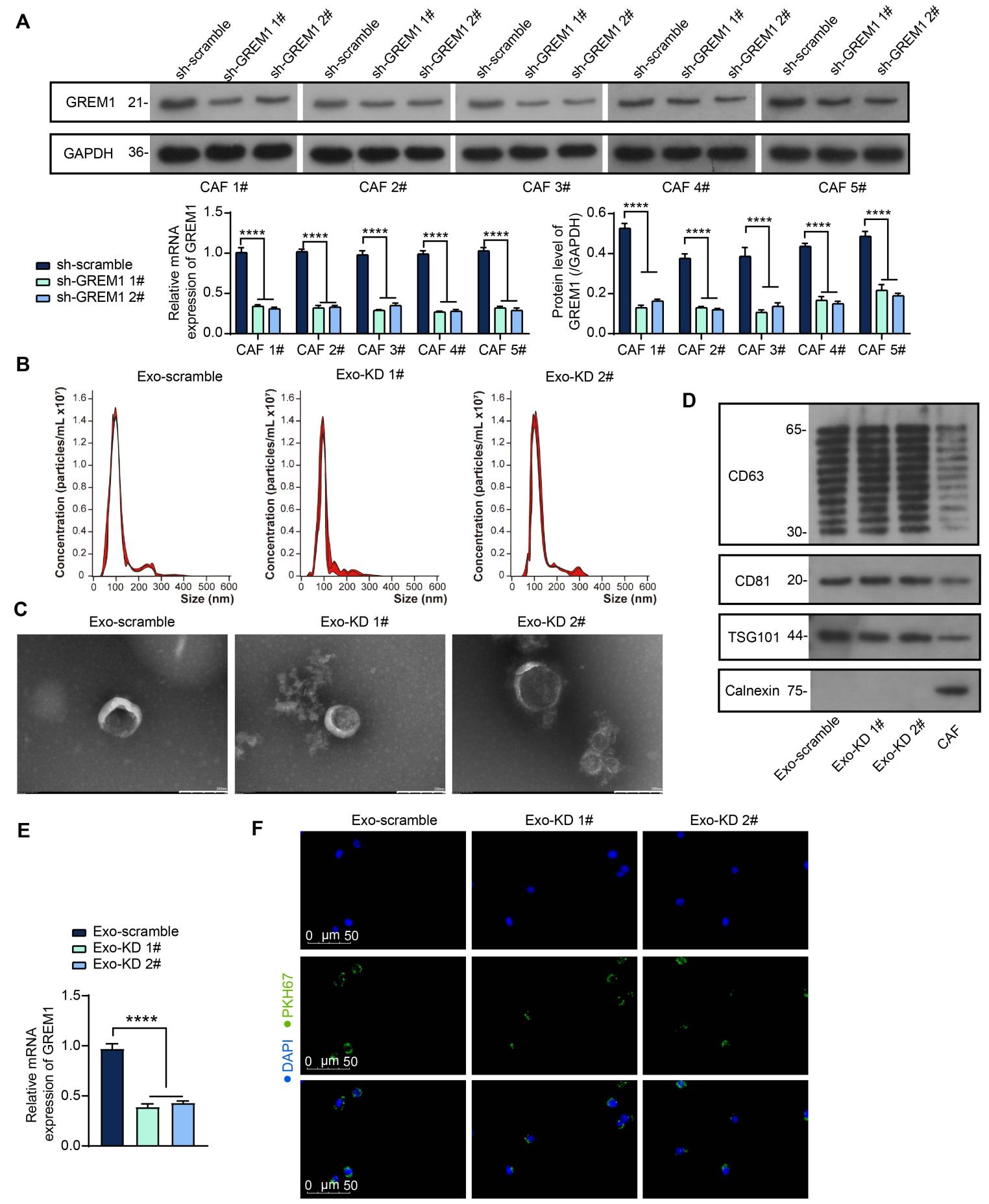
**

**Supplementary Figure 4** Characterization of exosomes derived from CAF with knockdown of GREM1. (A) The knockdown efficiency of shRNAs for GREM1 in CAF was analyzed using RT-qPCR and western blot analysis. (B) The particle size of exosomes obtained from CAFs transfected with shRNAs for GREM1 (Exo-KD 1# and Exo-KD 2#) was examined using NTA. (C) Morphologic structure of Exo-scramble, Exo-KD 1#, and Exo-KD 2# observed by TEM. (D) Expression of marker proteins for exosomes in CAF, Exo-scramble, Exo-KD 1#, and Exo-KD 2# was detected by western blot. (E) GREM1 mRNA in Exo-scramble, Exo-KD 1#, and Exo-KD 2# by RT-qPCR. (F) Uptake of Exo-scramble, Exo-KD 1#, and Exo-KD 2# by macrophages observed by PKH67 fluorescent labeling. The results are representative of data from triplicate experiments. The data are shown as mean ± SEM. *****p* < 0.001 (ANOVA).

**Supplementary Figure 5**

**
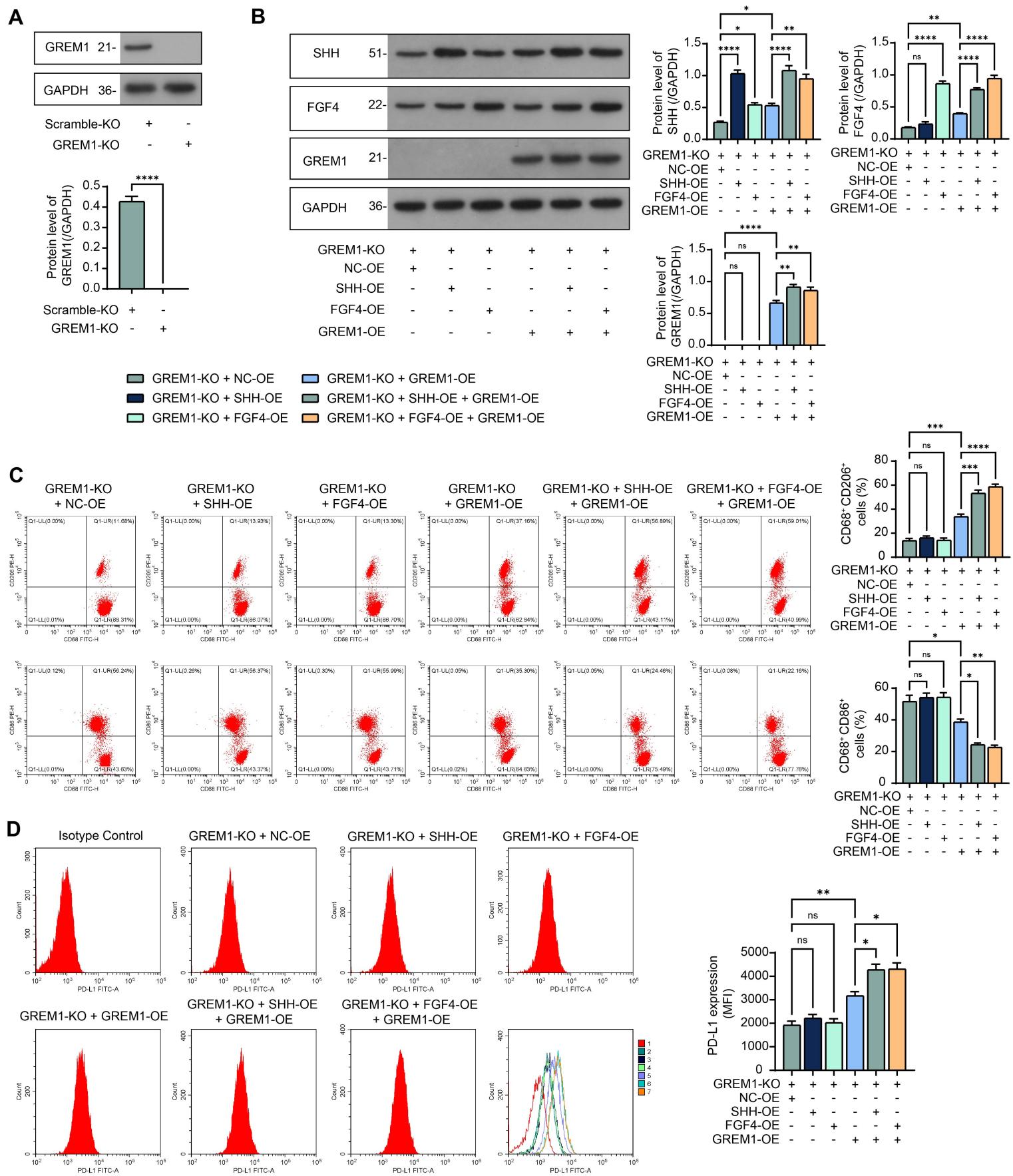
**

**Supplementary Figure 5** The promotion of the macrophage immunosuppressive phenotype by FGF4/SHH is dependent on GREM1 expression. (A) The successful establishment of macrophages with GREM1 knockout (GREM1-KO) was verified using the western blot assay. (B) The protein expression of FGF4, SHH, and GREM1 in GREM1-KO macrophages after transfection of an exogenous overexpression plasmid of FGF4/SHH/GREM1 was verified using western blot assay. (C) Flow cytometry analysis of the proportion of macrophages with M1 (CD68^+^CD86+) or M2 (CD68^+^CD206^+^) phenotype. (D) Detection of PD-L1 expression in macrophages by flow cytometry. The results are representative of data from triplicate experiments. The data are shown as mean ± SEM. **p* < 0.05, ***p* < 0.01, ****p* < 0.001, *****p* < 0.001 (t-test or ANOVA).
